# A deep learning method to predict bacterial ADP-ribosyltransferase toxins

**DOI:** 10.1093/bioinformatics/btae378

**Published:** 2024-06-17

**Authors:** Dandan Zheng, Siyu Zhou, Lihong Chen, Guansong Pang, Jian Yang

**Affiliations:** NHC Key Laboratory of Systems Biology of Pathogens, National Institute of Pathogen Biology, Chinese Academy of Medical Sciences and Peking Union Medical College, Beijing 102629, China; NHC Key Laboratory of Systems Biology of Pathogens, National Institute of Pathogen Biology, Chinese Academy of Medical Sciences and Peking Union Medical College, Beijing 102629, China; NHC Key Laboratory of Systems Biology of Pathogens, National Institute of Pathogen Biology, Chinese Academy of Medical Sciences and Peking Union Medical College, Beijing 102629, China; School of Computing and Information Systems, Singapore Management University, Singapore 178902, Singapore; NHC Key Laboratory of Systems Biology of Pathogens, National Institute of Pathogen Biology, Chinese Academy of Medical Sciences and Peking Union Medical College, Beijing 102629, China

## Abstract

**Motivation:**

ADP-ribosylation is a critical modification involved in regulating diverse cellular processes, including chromatin structure regulation, RNA transcription, and cell death. Bacterial ADP-ribosyltransferase toxins (bARTTs) serve as potent virulence factors that orchestrate the manipulation of host cell functions to facilitate bacterial pathogenesis. Despite their pivotal role, the bioinformatic identification of novel bARTTs poses a formidable challenge due to limited verified data and the inherent sequence diversity among bARTT members.

**Results:**

We proposed a deep learning-based model, ARTNet, specifically engineered to predict bARTTs from bacterial genomes. Initially, we introduced an effective data augmentation method to address the issue of data scarcity in training ARTNet. Subsequently, we employed a data optimization strategy by utilizing ART-related domain subsequences instead of the primary full sequences, thereby significantly enhancing the performance of ARTNet. ARTNet achieved a Matthew’s correlation coefficient (MCC) of 0.9351 and an *F*1-score (macro) of 0.9666 on repeated independent test datasets, outperforming three other deep learning models and six traditional machine learning models in terms of time efficiency and accuracy. Furthermore, we empirically demonstrated the ability of ARTNet to predict novel bARTTs across domain superfamilies without sequence similarity. We anticipate that ARTNet will greatly facilitate the screening and identification of novel bARTTs from bacterial genomes.

**Availability and implementation:**

ARTNet is publicly accessible at http://www.mgc.ac.cn/ARTNet/. The source code of ARTNet is freely available at https://github.com/zhengdd0422/ARTNet/.

## 1 Introduction

ADP-ribosylation is a ubiquitous modification of biomolecules found across all domains of life and known to regulate a variety of fundamental processes, such as chromatin structure, RNA transcription, cell differentiation, the antiviral response, energy metabolism, and cell death (Manco *et al.* 2022, Suskiewicz *et al.* 2023). This modification occurs through the transfer of a single or multiple ADP-ribose unit(s) from NAD+ onto target substrates by the release of nicotinamide by ADP-ribosyltransferase (ART) superfamilies. Bacterial ADP-ribosyltransferase toxins (bARTTs) are potent bacterial virulence factors that disrupt host cell functions by transferring single ADP-ribose to various eukaryotic substrates, thereby promoting bacterial pathogenesis (Simon *et al.* 2014, Bullen *et al.* 2022). Historically, bARTTs were known as post-translational modifications of proteins including heterotrimeric G proteins, Rho proteins, and actin (Aktories *et al.* 1986, 1989, Gill and Meren 1978). However, studies in recent years have demonstrated that nucleic acids can also be substrates of reversible ADP-ribosylation (Groslambert *et al.* 2021, Suskiewicz *et al.* 2023). For instance, reversible ADP-ribosylation of DNA on thymidine and guanosine bases occurs in cellulo through DarT of the bacterial toxin–antitoxin (TA) system DarTG, which is widespread among prokaryotes including many human pathogens and shown to provide control of DNA replication and bacterial growth as well as protection against bacteriophages (Schuller *et al.* 2021, 2023). In addition, Tre23, the C-terminal toxin domain of Rhs1 secreted by *Photorhabdus laumondii*, inhibits translation through ADP-ribosylation of 23S ribosomal RNA (Jurėnas *et al.* 2021). Similarly, RhsP2, an antibacterial toxin, secreted by *Pseudomonas aeruginosa*, ADP-ribosylates the 2′-hydroxyl groups of double-stranded RNA and tRNAs, leading to cellular intoxication (Bullen *et al.* 2022). These findings suggest that ADP-ribosylation of nucleic acids is a common yet largely unexplored aspect of ADP-ribosylation signaling, which may become an exciting area in the fields of DNA damage response, epigenetics, and beyond (Schuller *et al.* 2021).

More than 40 bARTTs have been reported, as shown in Supplementary Table S1. They are encoded by various important human pathogens, such as *Vibrio cholerae*, *Bordetella pertussis*, *Salmonella typhi*, *Staphylococcus aureus*, *P. aeruginosa*, *Mycoplasma pneumoniae*, *Corynebacterium diphtheriae*, and *Clostridium botulinum*. According to their toxin domain and conserved active site motifs, bARTTs are divided into two primary groups: diphtheria-like (DT-like) toxins with H-Y-E motifs and cholera-like (CT-like) toxins with R-S-E motifs (Rosado and Pioli 2021). DT-like toxins are single-chain AB toxins, with an A domain mediating the enzymatic activity responsible for halting protein synthesis in the target cell and a B domain binding to a cell receptor and mediating the translocation of the A chain into the cytosol. CT-like toxins are normally AB_5_ toxins with an A domain and B oligomer comprised of five noncovalently associated proteins (Sixma *et al.* 1993). CT-like toxins have three other derivatives: C2-like, C3-like, and CT-PT-like toxins (Fieldhouse *et al.* 2010). C2-like toxins are composed of an enzymatic component C2-I and a binding and translocation component C2-II (Schleberger *et al.* 2006). C3-like toxins are single-chain proteins consisting solely of a catalytic A subunit (Han *et al.* 2001). In addition, some recently discovered bARTTs have different structural organizations. For instance, typhoid toxin exhibits a unique A_2_B_5_ stoichiometry, with two covalently bonded A subunits (PltA and CdtB) linked to a pentameric B subunit composed of PltB or PltC (Fowler *et al.* 2019, Chang *et al.* 2022). Tc toxins are ABC toxins consisting of the binding component TcA, the functional linker component TcB, and the enzyme component TcC (Pfaumann *et al.* 2015, Belyy *et al.* 2022). Although many investigations on the role of bARTTs in pathological processes have been conducted during the last few decades, our understanding of the molecular mechanisms and cellular functions they mediate remains insufficient (Bullen *et al.* 2022). This gap in knowledge may result in a lack of understanding of numerous potentially related pathogenic mechanisms and disease pathways (Palazzo *et al.* 2019). Early efforts to identify bARTTs were based on genetics, cell biology, and biochemical analyses, which are very time-consuming (Simon *et al.* 2014). Subsequently, sequence similarity-based bioinformatics techniques such as BLAST or PSI-BLAST enabled the discovery of some homologous bARTT variants (Fieldhouse *et al.* 2010). However, despite bARTTs having a conserved structural organization of the core fold, most members exhibit significant sequence divergence (Weixler *et al.* 2021). Indeed, the upper quartile and median pairwise sequence similarities of the ART domain of 44 reported bARTTs were 19% and 16.9%, respectively (Supplementary Fig. S1), indicating that it is difficult, if not impossible, to identify novel bARTTs based on sequence similarity.

Deep learning (DL) has been widely applied in computational biology in recent years (Baek *et al.* 2021). Our previous work showed that a convolutional neural network (CNN) demonstrated the desired generalization performance for the classification of bacterial virulence factors by capturing conserved regions or motifs related to protein families (Zheng *et al.* 2020). Motivated by its success, in this study, we developed a CNN-based model, termed ARTNet, to address the issues described above that hinder *in silico* prediction of bARTTs. One key challenge is that the number of verified bARTTs is extremely limited, which is not conducive to the construction of DL models. To address this challenge, first and foremost, we introduced a data augmentation method based on the ART functional domain and generated a significantly expanded dataset, providing an important benchmark for developing novel methods (such as training deep methods) for the prediction of bARTTs. Then, we constructed ARTNet models based on the full-length sequence-based dataset and illustrated the effectiveness of our data augmentation approach. Considering that the full sequences of bARTTs typically contain irrelevant or noisy subsequences, we generated a more effective ARTNet that is trained using ART domain subsequences rather than the primary full sequences. Impressively, this optimization strategy significantly improved the performance of ARTNet, obtaining an MCC of 0.9351 and an *F*1-score (macro) of 0.9666 on repeated independent test datasets and outperforming three other DL models and six traditional machine learning (ML) models in terms of time efficiency and classification performance. This provides a new avenue for computational studies on related biological issues. Additionally, we empirically demonstrated the ability of ARTNet to predict *bona fide* novel bARTTs across domain superfamilies without sequence similarity. To facilitate the future application of ARTNet for bARTT prediction, we further developed a user-friendly online web server that is publicly accessible at http://www.mgc.ac.cn/ARTNet/.

## 2 Materials and methods

### 2.1 Data collection and processing

#### 2.1.1 Sequence-based data construction

**Figure 1.**
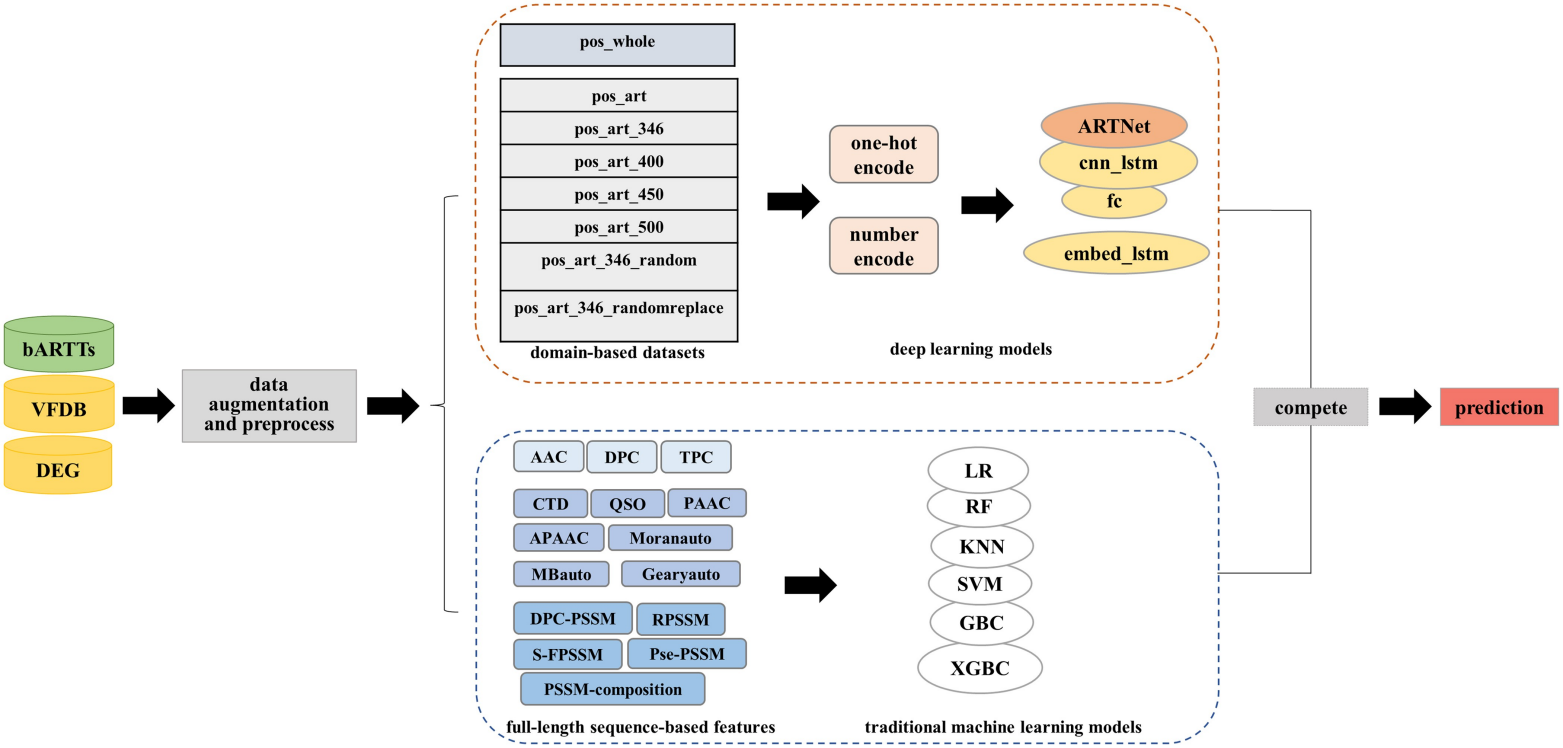
The overall workflow of the bacterial ADP-ribosyltransferase toxin prediction development method.

Figure 1 shows the entire workflow of the ARTNet approach. We first collected 44 reported experimentally verified bARTTs encoded by 27 different bacterial pathogens (Supplementary Fig. S2) to construct the original positive sample set. The core dataset of the virulence factor database (VFDB) (Liu *et al.* 2022), excluding the 44 known bARTTs, and the bacterial catalog of the database of essential genes (DEG) (Luo *et al.* 2021) were merged to construct the negative sample set. A limited quantity of positive samples may prevent DL or ML models from learning sufficient valuable features to build prediction systems. Further analyses revealed that the ART-related functional domains of the 44 known bARTTs were mainly categorized into three superfamilies: ‘ADP_ribosyl’ (cl00283), ‘VIP2’ (cl00173), and ‘Enterotoxin_a’ (cl03779) (Supplementary Table S1). The ADP_ribosyl domain presents in diphtheria toxin from *C. diphtheriae*, which inhibits protein synthesis by transferring ADP-ribose from NAD+ to elongation factor 2 (Bennett and Eisenberg 1994, Baldi and Sadowski 2014). ExoS secreted by *P. aeruginosa* encodes the VIP2 domain that ADP-ribosylates numerous host proteins, resembling vertebrate mono-ARTs (Van der Maaten and Hinton 2008). Pertussis toxin from *B. pertussis* carries an Enterotoxin_a domain that ADP-ribosylates inhibitory alpha-subunits of G proteins to disrupt G protein-coupled receptor signaling (Katada *et al.* 1983, Carbonetti 2010). Motivated by these experimental evidences, we downloaded all protein sequences related to these three domain superfamilies available from GenBank (accessed in April 2021) and predict their conserved domain via CD-Search (Lu *et al.* 2020). We extracted 41 267 sequences with conserved domain that exactly matched the three superfamilies to construct an expanded positive sample set. Then, we deleted invalid sequences, including duplicate samples, nonstandard amino acid-containing samples, and very short samples (<50 amino acids), and further removed redundant samples of high homology by CD-HIT (Fu *et al.* 2012) (90%) to produce an expanded positive dataset of 3158 sequences. The negative sample set mentioned above was also refined with the same processes and cutoffs, which yielded a collection of 19 653 sequences.

We randomly selected 10 bARTTs from the original positive sample set, 314 sequences from the expanded positive sample set (1/10 of each superfamily), and 324 sequences from the negative sample set to perform an independent test. The remaining sequences of the expanded positive set, designated as pos_whole, were used as training data. For the construction of the DL models, we applied a slide window with a size of 1000 (step = 1) to truncate long sequences of pos_whole to satisfy the equal length input and used CD-HIT (70%) to remove redundancy. This data partitioning process was repeated five times. More details of the data preprocessing pipeline are illustrated in Supplementary Fig. S3.

#### 2.1.2 Domain-based data construction

To enable classifiers to accurately learn the features of ART-related domains, we also carried out domain-based data optimization. Specifically, the subsequences of known or predicted ART-related domains of the samples in the original positive sample set and the expanded positive sample set described above were extracted and represented as pos_art to train the DL models. However, we realized that DL models trained on pure ART-related domains were prone to overfitting and failed to identify real-world samples with irrelevant noise (data not shown). We, therefore, constructed several variants of pos_art by including upstream and downstream contexts based on their original full-length sequences, which was found to be effective in alleviating this issue. In particular, we first extended each subsequence of the ART-related domain from the middle to 346 amino acids (the maximum length of pos_art) or a longer length, including 400, 450, and 500 amino acids, to produce positive sample sets, designated as pos_art_346, pos_art_400, pos_art_450, and pos_art_500, respectively. Second, to determine the effect of context, we shifted the extended amino acids of pos_art_346 in two ways: (1) randomly shuffling the order of the extended amino acids at each end to produce pos_art_346_random and (2) randomly replacing each amino acid with any of the 20 standard amino acids to produce pos_art_346_randomreplace. These datasets were refined with the same processes and cutoffs as those described above. Sequences identical to those in the independent test were excluded, and the remaining sequences were used as training data. One issue was that these domain-based positive datasets had different sample length distributions than the negative dataset (Supplementary Fig. S4), which may have created an undesired artifact for the model to learn. Therefore, we utilized a sliding window strategy (step = 1) with a size the same as the maximum length of each domain-based positive dataset, which helped truncate full-length negative samples to fit similar length distributions. CD-HIT (70%) was then used to remove redundancy in the truncated negative sample set. The statistical details of the datasets described above can be found in Supplementary Table S2.

### 2.2 ARTNet: our proposed DL model

We proposed a CNN-based model, designated ARTNet, to predict bARTTs. ARTNet applied an end-to-end prediction procedure that began with protein sequences in FASTA format and ended with the predicted classification of bARTTs. It included one input layer, two 1D convolutional layers (Conv1D), two global max pooling (Maxpooling1D) layers, one fully connected (fc) layer, and one prediction/output layer. Formally, we had:

$$y_{i}=g\left(f\left(x_{i}\right)\right),$$

where$\;x_{i}$ represents the input protein sequence, f is the feature representation learner consisting of all the layers before the prediction layer, and g is the prediction layer used to predict the input sequence.

Specifically, every input sequence was transformed into a one-hot encoding matrix based on its appearance in the alphabet (Zheng *et al.* 2020). A zero-padding strategy was applied to align the input length (Taghouti *et al.* 2016). If $X_{i}$ represented the one-hot encoding matrix of $x_{i}$, the feature representation learner could be represented as follows:

$$f=f^{\mathrm{fc}}\;○\;f^{\mathrm{con}}\left(X_{i};\Theta_{f}\right),$$

where $f^{\mathrm{fc}}$ represents one fc mapping function, ‘○’ is a compound operation, $\Theta_{f}$ is the set of parameters to be learned, and $f^{\mathrm{con}}$ consists of two nonlinear convolution and pooling operations, each of which can be defined as follows:

$$f^{\mathrm{con}}=\mathrm{Maxpoolong}1D\left(\mathrm{Conv}1D\left(\mathrm{Maxpoolong}1D\left(\mathrm{Conv}1D\left(X_{i}\right)\right)\right)\right).$$

We set the first Conv1D with a filter number of 256 and kernel size of 9 and the second Conv1D with a filter number of 128 and kernel size of 7. The rectified linear unit (ReLU) function was used as a nonlinear activation function in each convolutional layer to transform the data from one volume to another (Veltri *et al.* 2018). We set the Maxpooling1D size to 5 to reduce the output dimension of Conv1D. The fc layer with 128 units was applied after convolution to learn more expressive high-level abstract features. We applied a dropout (0.5) after the pooling layer and the fc layer to avoid overfitting by randomly masking the positions of the output (Baldi and Sadowski 2014). The prediction layer contained a single neuron and applied the sigmoid function to produce the prediction probability for $y_{i}=1$, defined as ${p(y}_{i}=1|x_{i})$. We set 0.5 as the prediction threshold, and a prediction value greater than 0.5 was considered positive. Binary cross-entropy loss and the Adam (Kingma and Ba 2014) optimizer were used to determine the parameters of the models. The learning rate was set to 0.001 by default, and the batch size and the number of epochs were set to 128 and 100, respectively. We tested a range of convolution options, including 64, 128, and 256 for the filter size, 5, 7, and 9 for the kernel size, 3 and 5 for the max pooling size, and 64, 128, and 256 for the batch size. Finally, we fixed these hyperparameters based on 5-fold cross-validation results (data not shown). The Keras (http://www.keras.io) library with a TensorFlow (http://tensorflow.org/) backend in Python was used to implement DL models, which were executed with four GeForce RTX 2080 Ti graphics cards.

### 2.3 Other competing DL models

To develop a more accurate and efficient bARTT prediction model and examine the effectiveness of ARTNet, we constructed three additional DL models, namely, ‘onehot+cnn_lstm’, ‘onehot+fc’, and ‘number+embed’. Specifically, ‘onehot+cnn_lstm’ replaced the second convolutional layer of ARTNet with long short-term memory (LSTM) (LeCun *et al.* 2015) (128 units), while the ‘onehot+fc’ network replaced two convolutional layers with two fc layers (256 and 128 units, respectively). Instead of using one-hot encoding, ‘number+embed’ converted the peptide sequence into a zero-padded numeric vector using numbers 1–20 to represent each of the 20 standard amino acids (Veltri *et al.* 2018) and fed them to an embedding layer (128 units), a convolutional layer and an LSTM layer to perform feature abstraction. More structural details are listed in Supplementary Fig. S5.

### 2.4 Traditional ML models using predefined features

Traditional ML algorithms with predefined features have demonstrated good performances for predicting virulence factors from entire sequences (Xie *et al.* 2021). To verify the advantages of our proposed ARTNet, we applied six well-established classification algorithms, namely, logistic regression (LR), support vector machine (SVM), k-nearest neighbors (KNN), random forest (RF), gradient boosting classifier (GBC), and extreme gradient boosting classifier (XGBC), as the baselines (Zeng and Zou 2019, Xu *et al.* 2021). Building stable, dependable classifiers with competitive performance requires efficient feature extraction (Xie *et al.* 2021). To thoroughly study the typical and particular patterns of bARTT proteins, we extracted 15 widely used predefined features, including three major groups: a sequence-based features group [AAC (Anfinsen 1972), DPC (Zou *et al.* 2013), and TPC (Chou 2000, Hosen *et al.* 2022)], a physicochemical property-based features group [CTD (Cao *et al.* 2013), QSO (Chou 2000), PAAC (Chou 2001), APAAC (Chou 2001), MBauto (Lin and Pan 2001), Moranauto (Horne 1988), and Gearyauto (Sokal and Thomson 2006)] and an evolutionary information-based features group [PSSM-composition (Zou *et al.* 2013), S-FPSSM (Zahiri *et al.* 2013), DPC-PSSM (Liu *et al.* 2010), Pse-PSSM (Chou and Shen 2007), and RPSSM (Chen *et al.* 2023)]. Sequence-based features describe the frequencies or compositions of sequence elements, whereas physicochemical property-based features represent the statistical information about the physicochemical properties of the amino acids in protein sequences. We applied the propy program (Cao *et al.* 2013) for their extraction. Previous studies have demonstrated that the evolutionary information of sequences can sometimes be more insightful than that of sequences (Wang *et al.* 2011, 2018, 2019, An *et al.* 2018). We applied a PSI-BLAST search against UniRef50 (accessed in May 2023) with the parameters *j* = 3 and *e*-value = 0.001 to obtain the original PSSM profiles and used POSSUM (Wang *et al.* 2017) to generate PSSM profile-based features. More details can be found in the Supplementary Methods.

### 2.5 Performance assessment

We applied 5-fold cross-validation to train models by dividing train data into training and validation datasets at a ratio of 4:1 and compared the models on five repeated independent test datasets. The reported performance was averaged over the results of the five implementations. Accuracy, precision, recall, *F*1-score, and MCC were used to evaluate the performance of all methods, and their formulas are listed below:

$$\begin{array}{c} a\mathrm{ccuracy}=\frac{TP+TN}{TP+TN+FP+FN},\;\\ \begin{array}{c} p\mathrm{recision}=\frac{TP}{TP+FP},\;\\ \begin{array}{c} r\mathrm{ecall}=\frac{TP}{TP+FN\;},\;\\ \begin{array}{c} F1-\mathrm{score}=\frac{2*(\mathrm{precision}*\mathrm{recall})}{\mathrm{precision}+\mathrm{recall}},\\ MCC=\frac{TP*TN-FP*FN}{\sqrt{\left(TP+FP)(TP+FN)(TN+FP)(TN+FN\right)}},\; \end{array} \end{array} \end{array} \end{array}$$

where TP, TN, FP, and FN denote the numbers of true positives, true negatives, false positives, and false negatives, respectively. The MCC ranged from −1 to 1, with a higher MCC indicating better performance. In addition, the receiver operating characteristic (ROC) curve and the precision–recall (PR) curve were plotted to visualize the comprehensive performance of the model. The area under the ROC curve (AUC) and the area under the PR curve (AP) were also calculated to quantify the respective performances. The higher the area value is, the better the prediction performance.

## 3 Results

### 3.1 Construction of ARTNet on the sequence-based dataset

We first constructed our proposed ARTNet on pos_whole and then applied it to predict independent test samples. Each target sequence in the independent test was truncated to a length threshold of 1000 to align with the input dimensions of the model. ARTNet demonstrated good performance on the validation samples (Table 1) and performed well on repeated independent test datasets, with an MCC of 0.9004 and an *F*1-score (macro) of 0.9490 (Table 2). To investigate whether the data expansion procedure effectively improved the performance of ARTNet as expected, we excluded the expanded positive samples in both train and independent test datasets and compared the performances of ARTNet before and after data expansion. Undoubtedly, before data expansion, ARTNet performed poorly, with an MCC of approximately 0.5 on both the validation and independent test samples. When focusing on the 10 verified bARTTs in the independent test datasets, we found that the mean accuracy of ARTNet significantly improved from 44.44% to 100%, benefiting from the data expansion.

**Table 1.** Performance (mean ± SD) of ARTNet combined with pos_whole on repeated 5-fold cross-validation before and after data augmentation.

Method	Accuracy	Sensitivity	Specificity	*F*1-score (micro)	Precision (macro)	Recall (macro)	*F*1-score (macro)	MCC
pos_whole	0.9816 (±0.0016)	**0.9447 (**±**0.0021)**	0.9909 (±0.0023)	0.9816 (±0.0016)	**0.9748 (**±**0.0042)**	**0.9678 (**±**0.0010)**	**0.9712 (**±**0.0024)**	**0.9425 (**±**0.0049)**
before_data_augmentation	**0.9955 (**±**0.0050)**	0.4686 (±0.1465)	**0.9964 (**±**0.0050)**	**0.9955 (**±**0.0050)**	0.8487 (±0.0772)	0.7325 (±0.0732)	0.7270 (±0.0551)	0.5072 (±0.0954)

*Note*: Expanded positive samples in train and independent sets were excluded in ‘before_data_augmentation’. The best indicators are shown in bold.

**Table 2.** Performance (mean ± SD) of ARTNet combined with pos_whole on repeated independent test datasets before and after data augmentation.

Method	Accuracy	Sensitivity	Specificity	*F*1-score (micro)	Precision (macro)	Recall (macro)	*F*1-score (macro)	MCC
pos_whole	0.9491 (±0.0070)	**0.9149 (**±**0.0115)**	0.9832 (±0.0031)	0.9491 (±0.0070)	**0.9513 (**±**0.0064)**	**0.9491 (**±**0.0070)**	**0.9490 (**±**0.0070)**	**0.9004 (**±**0.0134)**
before_data_augmentation	**0.9746 (**±**0.0071)**	0.3120 (±0.1418)	**0.9951 (**±**0.0071)**	**0.9746 (**±**0.0071)**	0.8944 (±0.0604)	0.6535 (±0.0701)	0.7005 (±0.0872)	0.4699 (±0.1570)

*Note*: Expanded positive samples in train and independent sets were excluded in ‘before_data_augmentation’. The best indicators are shown in bold.

### 3.2 Construction of ARTNet on domain-based datasets

To enhance the predictive performance of ARTNet, we first constructed pos_art, the ART-related domain-based dataset, to train ARTNet via 5-fold cross-validation and evaluated it on independent test datasets. Table 3 shows that on the validation samples, pos_art outperformed pos_whole, with a nearly 3% improvement in sensitivity, suggesting that short and precise subsequences made classification easier than long subsequences or full-length sequences did. However, on an independent test, ARTNet trained on pos_whole (with a length threshold of 1000) outperformed ARTNet trained on pos_art (with a length threshold of 346) by 54% in terms of sensitivity and 44% in terms of the MCC (Supplementary Table S3). By examining the differences between training and independent samples, we discovered that ARTNet trained on pos_art could identify subsequences composed of pure domains but failed to predict subsequences within the upstream and downstream context, implying that the model was overfitting.

**Table 3.** Performance (mean ± SD) of ARTNet combined with eight datasets on repeated 5-fold cross-validation.

Method	Accuracy	Sensitivity	Specificity	*F*1-score (micro)	Precision (macro)	Recall (macro)	*F*1-score (macro)	MCC
pos_art	0.9937 (±0.0016)	0.9745 (±0.0032)	0.9952 (±0.0017)	0.9937 (±0.0016)	0.9713 (±0.0082)	0.9849 (±0.0019)	0.9777 (±0.0052)	0.9559 (±0.0098)
pos_art_346	0.9901 (±0.0018)	0.9237 (±0.0064)	0.9961 (±0.0019)	0.9901 (±0.0018)	0.9757 (±0.0091)	0.9599 (±0.0033)	0.9674 (±0.0053)	0.9353 (±0.0104)
pos_art_400	0.9892 (±0.0009)	0.9262 (±0.0020)	0.9958 (±0.0011)	0.9892 (±0.0009)	0.9761 (±0.0051)	0.9610 (±0.0007)	0.9683 (±0.0024)	0.9369 (±0.0049)
pos_art_450	0.9886 (±0.0010)	0.9251 (±0.0022)	0.9959 (±0.0012)	0.9886 (±0.0010)	0.9771 (±0.0050)	0.9605 (±0.0010)	0.9685 (±0.0026)	0.9373 (±0.0052)
pos_art_500	0.9877 (±0.0015)	0.9218 (±0.0071)	0.9957 (±0.0020)	0.9877 (±0.0015)	0.9772 (±0.0076)	0.9588 (±0.0031)	0.9676 (±0.0037)	0.9357 (±0.0074)
pos_art_346_random	0.9956 (±0.0002)	0.9922 (±0.0003)	**0.9990 (**±**0.0001)**	0.9956 (±0.0002)	0.9957 (±0.0002)	0.9956 (±0.0002)	0.9956 (±0.0002)	0.9912 (±0.0004)
pos_art_346_randomreplace	**0.9984 (**±**0.0002)**	**0.9981 (**±**0.0003)**	0.9988 (±0.0001)	**0.9984 (**±**0.0002)**	**0.9984 (**±**0.0002)**	**0.9984 (**±**0.0002)**	**0.9984 (**±**0.0002)**	**0.9968 (**±**0.0003)**
pos_whole	0.9816 (±0.0016)	0.9447 (±0.0021)	0.9909 (±0.0023)	0.9816 (±0.0016)	0.9748 (±0.0042)	0.9678 (±0.0010)	0.9712 (±0.0024)	0.9425 (±0.0049)

*Note*: The best indicators are shown in bold.

To address this issue, we carried out data optimization by introducing various levels of noise based on the original full-length sequences and constructed six additional domain-based datasets (details described in Section 2). It should be noted that the datasets pos_art_346, pos_art_400, pos_art_450, and pos_art_500 had fewer positive training samples than pos_whole due to efficient redundancy removal of short sequences, while pos_art_346_random and pos_art_346_randomreplace had many more positive training samples than pos_whole due to expansion by domain context randomization (Supplementary Table S2).

**Figure 2.**
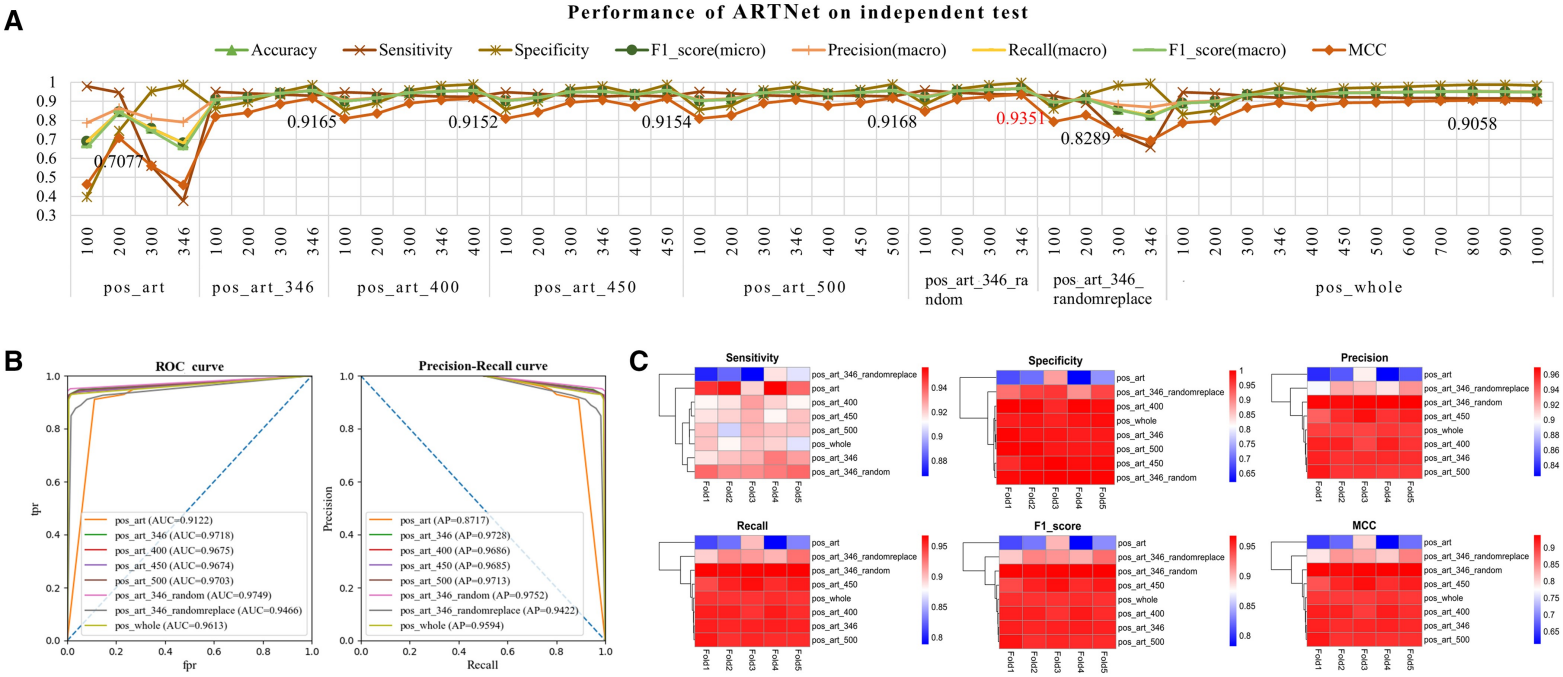
Performance of ARTNet combined with different data preprocessing methods on repeated independent test datasets. (A) Performance comparison of ARTNet combined with eight datasets using different length thresholds. The MCC value of each method is labeled. (B) ROC curves and precision-recall curves of ARTNet on one of five repeated independent tests. Only the results of the best length threshold of each model are plotted. (C) Heatmap of the sensitivity, specificity, precision (macro), recall (macro), *F*1-score (macro), and MCC of the eight data preprocessing methods. Only the results of the best length threshold of each model are plotted. Fold1–5 refer to the five models produced by 5-fold cross-validation in one of five repeated experiments.

We trained ARTNet on these datasets individually using an input length dimension of model structure equal to the maximum sequence length of the corresponding dataset. Table 3 indicates that when the ART-related domain was extended to lengths of 346, 400, 450, or 500, the MCC declined nearly 2% compared with that of pos_art, but when the context of the ART-related domain was randomized (pos_art_346_random and pos_art_346_randomreplace), all metrics exceeded 99%. We then applied the ARTNet models trained on these datasets to predict independent test datasets. Before prediction, we truncated each target sequence in an independent test with length thresholds ranging from 100 to 1000 to explore the best length parameter. As expected, the models trained with pos_art_346, pos_art_400, pos_art_450, pos_art_500, and pos_art_346_random outperformed those trained with pos_whole (1–3%) and pos_art (21–23%) in terms of the MCC when the best length threshold was used (Fig. 2A and Supplementary Table S3), suggesting that our domain-based data optimization improved the generalization ability of ARTNet. There were no significant differences among the performances of the models trained on pos_art_346, pos_art_400, pos_art_450, and pos_art_500; therefore, we only used pos_art_346 as a representative training sample set in our subsequent experiments. Among all datasets, pos_art_346_random demonstrated the best performance, with an MCC of 0.9351 and an *F*1-score (macro) of 0.9666 with a length threshold of 346. ROC curves and PR curves (Fig. 2B and Supplementary Fig. S6) indicated that ARTNet trained on pos_art_346_random achieved the best AUC and AP, exceeding 0.97, on almost all independent test sets. Notably, this dataset had more training data than the others, which demonstrated the effectiveness of our domain-based data optimization strategies and highlighted the importance of large datasets for model improvement. However, when the upstream and downstream information was completely destroyed rather than simply disrupted, pos_art_346_randomreplace did not show any advantages on the independent test datasets. Presumably, the locations of ART-related domains are not as accurate as expected, or alternatively, some unknown features within the context are critical for domain prediction. A thorough analysis indicated that ARTNet generated the best results for almost all datasets when using a length threshold similar to the model input length dimension, which was applied as the length threshold of the objective sequences in the following experiments, e.g. pos_art_346_random of 346, pos_art_346 of 346, and pos_whole of 1000.

In addition, to investigate the consistency of the ARTNet models, we used heatmaps to visualize the metrics predicted by the five models (from 5-fold cross-validation) on the corresponding independent test set (Fig. 2C). For each index, the values among the five models were generally very close. Moreover, the clustering of rows indicated that the performances of the ARTNet models trained on all datasets except for pos_art and pos_art_346_randomreplace were similar. We also generated a Venn diagram to analyze the ability of the five models trained on pos_art_346_random to predict 324 true-positive samples from an independent test set (Supplementary Fig. S7). These remarkably consistent results highlight the stability and robustness of ARTNet. In addition, we used VFDB and DEG individually as a negative set to further explore the impact of different negative datasets on ARTNet. Supplementary Table S4 shows that no significant difference was found between them according to 5-fold cross-validation. Therefore, we applied the combination of VFDB and DEG as a negative set in this work.

### 3.3 Comparison of ARTNet with other DL models

**Figure 3.**
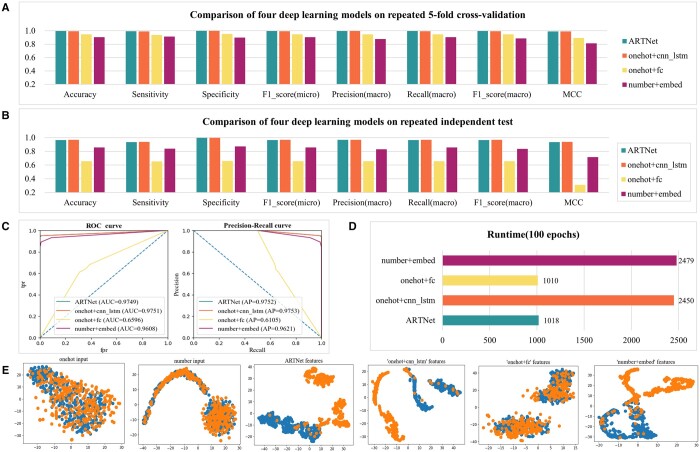
Comparison of four deep learning models combined with pos_art_346_random. (A) Comparison of four deep learning models on repeated 5-fold cross-validation. (B) Comparison of four deep learning models on repeated independent test datasets. (C) ROC curves and precision–recall curves of four deep learning models on one of five repeated independent tests. (D) Training time of four deep learning models per 100 epochs. (E) T-SNE visualization of two encoded input datasets and four model-learned features based on one of five repeated independent tests.

We constructed three other DL models on pos_art_346_random (optimum for ARTNet) to investigate the effectiveness of ARTNet. On both the repeated 5-fold cross-validation datasets (Fig. 3A and Supplementary Table S5) and the independent test datasets (Fig. 3B and Supplementary Table S6), ‘onehot+cnn_lstm’ was equivalent to ARTNet, while ‘onehot+fc’ and ‘number+embed’ were worse than ARTNet in terms of all the metrics. ROC curves and PR curves (Fig. 3C and Supplementary Fig. S8) also verified this result. The classification metrics between ARTNet and ‘onehot+cnn_lstm’ were not significantly different, but the training speed of ARTNet was twice as fast as that of ‘onehot+cnn_lstm’ (Fig. 3D). Undoubtedly, fast training is crucial for the development of DL models with large amounts of data. *T*-distributed stochastic neighbor embedding (t-SNE) (Van der Maaten and Hinton 2008) was further applied in an independent test to explore the underlying reasons for the differences in performance among the DL models. The raw input of one-hot encoding or numerical encoding was disorganized, but after feature abstractions (particularly ARTNet and ‘onehot+cnn_lstm’), the samples became clear and separable (Fig. 3E), which demonstrates the rationality of the ARTNet model structure.

**Figure 4.**
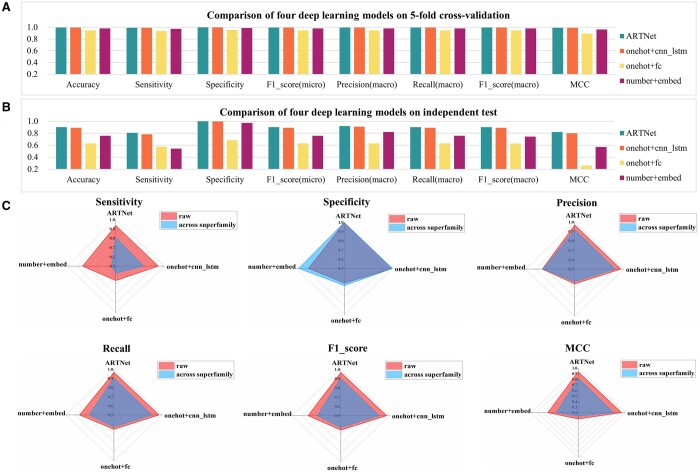
Comparison of the ability of four deep learning models combined with pos_art_346_random to predict proteins across superfamilies. (A) Comparison of four deep learning models on 5-fold cross-validation. (B) Comparison of four deep learning models on an independent test dataset. (C) Radar charts to compare the sensitivity, specificity, precision (macro), recall (macro), *F*1-score (macro), and MCC of four deep learning models on the independent test with or without across superfamilies. The ‘raw’ refers to the results that without across superfamilies.

To examine whether ARTNet has the ability to predict proteins across different domain superfamilies, we extracted all 358 sequences encoding the ‘ADP_ribosyl’ domain from the expanded positive sample set, along with 358 randomly selected sequences from the negative sample set, to build a new independent test. Then, the remaining samples, including 2800 sequences encoding either the ‘VIP2’ or ‘Enterotoxin_a’ domain from the expanded positive sample set and the remaining negative samples, were used to train the pos_art_346_random model as described above. On the validation samples (Fig. 4A and Supplementary Table S7), all the models demonstrated a good performance, as they exhibited similar characteristics from the training samples. On an independent test (Fig. 4B and Supplementary Table S8), ARTNet outperformed the others in terms of all metrics and showed a relatively strong ability to predict protein sequences of unseen superfamilies, with an MCC of 0.8214 and an *F*1-score (macro) of 0.9022. Radar charts (Fig. 4C) were generated to compare the results of independent tests with (Supplementary Table S8) or without (Supplementary Table S6) across superfamilies. Across all the models, except for specificity, there was a notable decrease in all the metrics, particularly sensitivity, which decreased by 8–30%. This suggested substantial variations in fundamental characteristics among these superfamilies, presenting a challenging classification task for DL models. We investigated whether pos_art_346 or pos_whole could help DL models predict proteins across superfamilies. Unfortunately, they appeared to lack such capabilities, especially pos_whole, where the MCC was only 0.14 (Supplementary Tables S9 and S10).

### 3.4 Comparison of ARTNet with traditional ML baseline methods

**Figure 5.**
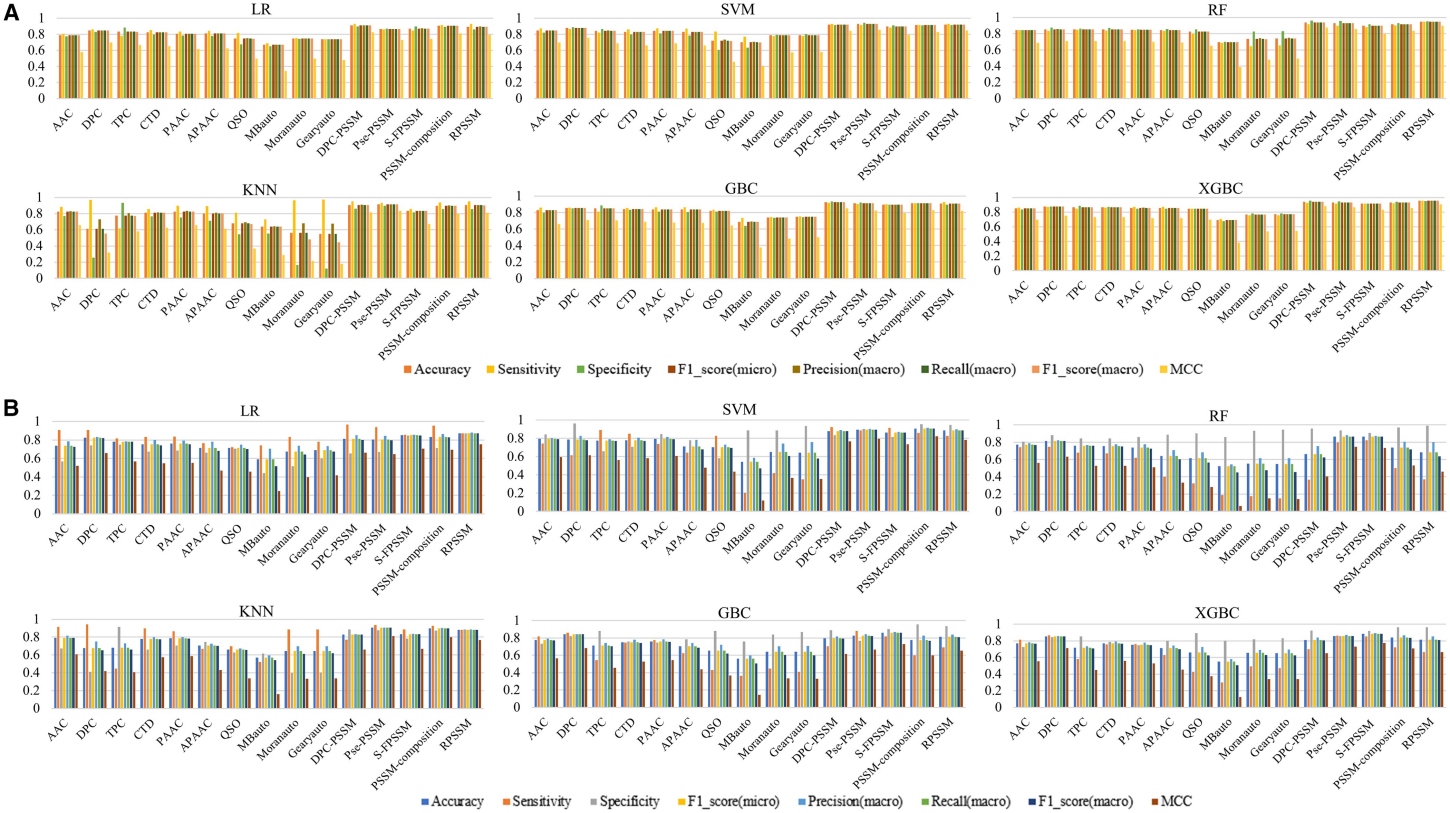
Comparison of six traditional machine learning models combined with 15 predefined features based on original full-length sequences. (A) Comparison of six traditional machine learning models combined with 15 predefined features on repeated 5-fold cross-validation. (B) Comparison of six traditional machine learning models combined with 15 predefined features on repeated independent test datasets.

**Figure 6.**
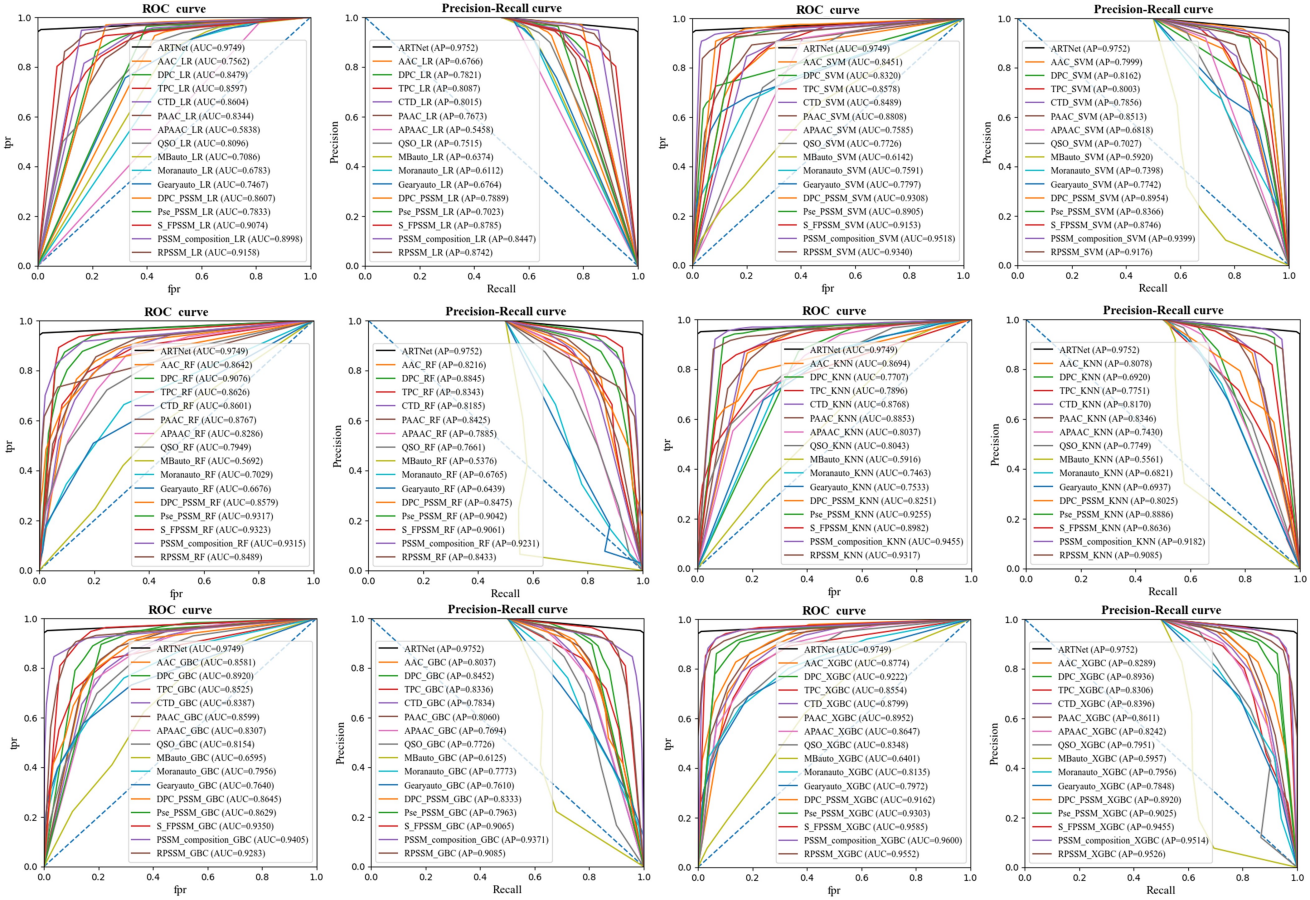
ROC curves and precision–recall curves of ARTNet combined with pos_art_346_random and six traditional machine learning models using 15 predefined features on one of five repeated independent tests.

To verify the advantages of ARTNet over traditional ML methods, we implemented six well-established ML classifiers combined with 15 predefined features to construct bARTT prediction models using protein sequences. Undersampling was used during model training for the class imbalance problem. On both 5-fold cross-validation (Fig. 5A and Supplementary Table S11) and independent tests (Fig. 5B and Supplementary Table S12), for almost all algorithms, features based on evolutionary information, e.g. DPC-PSSM, outperformed sequence-based features and physicochemical property-based features. Among all combinations of algorithms and features, SVM using PSSM-composition feature achieved the best results, with an MCC of 0.8221 on independent test datasets (Supplementary Table S12), but this value was still about 8–11% lower than that of ARTNet trained on pos_art_346_random, pos_art_346 or pos_whole (Supplementary Table S3). ROC curves and PR curves (Fig. 6 and Supplementary Fig. S9) showed that no combination on independent test datasets achieved an AUC or AP exceeding 0.96, while ARTNet trained on pos_art_346_random achieved over 0.97 in both AUC and AP performance, indicating that ARTNet outperformed all predefined features-based ML models.

### 3.5 Availability of the online bARTTs prediction service

To facilitate future application of ARTNet, we created a user-friendly online web server for the prediction of potential bARTTs from protein sequences of interest. The web server was written in Perl CGI and can be accessed for free at http://www.mgc.ac.cn/ARTNet/. Users can submit one or multiple sequences in FASTA format for prediction by a single click. In particular, as numerous previous studies have indicated that ensemble models are able to achieve significantly improved performance over the original baseline models (Wang *et al.* 2019, Xie *et al.* 2021, Yu *et al.* 2023, Liu *et al.* 2024), the ARTNet models trained on pos_art_346, pos_art_346_random, and pos_whole were used to build an ensemble method. To meet the demands of different users for further interpretation of the prediction results, the web server provides three modes, comprehensive, medium, and strict, to report positive sequences supported by at least one model, at least two models, and all three models, respectively. The tested computational time for a set of 1000 proteins is about 3 min. The maximum number of sequences in one batch was set to 5000 to avoid abuse and overloading. For privacy, the sequences uploaded by users and the corresponding prediction results will be deleted from the server three days after analysis. Users may download the prediction results for further local analyses in the future. Users can also download our source code to perform personalized large-scale sequence predictions from https://github.com/zhengdd0422/ARTNet/.

### 3.6 A case study

We conducted a case study based on two very recently verified bARTTs to examine the predictive scalability and robustness of our proposed approach. RhsP2 is an ART toxin exported by *P. aeruginosa* HSI-2 T6SS, which kills competitor cells through the ADP-ribosylation of structured noncoding RNAs (Bullen *et al.* 2022). Tre23 is an antibacterial toxin delivered by *P. laumondii* T6SS, which inhibits translation through ADP-ribosylation of 23S ribosomal RNA (Jurėnas *et al.* 2021). In particular, neither was included in our positive sample set since they were reported after our original data collection. In addition, both of them show little sequence similarity [<10% as computed by MatGat (Campanella *et al.* 2003)] with the ART-related domains from the 44 previously identified bARTTs (Supplementary Fig. S1). Nevertheless, our ARTNet server successfully predicted both RhsP2 and Tre23 as possible bARTTs in comprehensive mode. Indeed, the recent crystal structure of RhsP2 revealed two perpendicularly orientated β sheets that form the core of the toxin fold, resembling the catalytic domain of numerous ARTs, such as Exotoxin A from *P. aeruginosa* and diphtheria toxin from *C. diphtheriae* (Bullen *et al.* 2022). These results suggested the difficulty in identifying potential novel bARTTs using similarity-based methods and highlighted the usefulness and reliability of our proposed ARTNet. Furthermore, we also used ARTNet to predict the DarT toxin of TA system DarTG encoded by *Mycobacterium tuberculosis*, but not surprisingly, ARTNet did not predict successfully. Indeed, previous phylogenetic analysis of DarT showed that it was distinct from other bacterial diphtheria toxin-like ARTs and closer to eukaryotic members of poly(ADP-ribose)polymerase (PARP) (Jankevicius *et al.* 2016), and recent structure confirmed DarT as a diverged member of the PARP family (Schuller *et al.* 2021). Since our dataset comprises only bacterial bARTTs, identifying DarT proves challenging by the current model. This limitation guides our future research efforts.

## 4 Discussion

The bARTTs are potent bacterial virulence factors that orchestrate the manipulation of host cell functions to facilitate bacterial pathogenesis. More than 40 bARTTs have been reported to be encoded by a variety of important human pathogens, indicating the potential existence of additional undiscovered bARTTs that may play significant pathogenic roles in bacterial genomes. Most bARTTs exhibit significant sequence divergence, making it challenging, if not impossible, to identify novel bARTTs solely based on sequence similarity. In this work, we developed ARTNet, a DL-based model designed specifically for predicting bARTTs from bacterial genomes. To overcome the issue of the limited number of positive samples, we implemented effective data augmentation according to ART-related functional domains encoded by full-length protein sequences. While this similarity-based approach may introduce potential false positives, it significantly contributed to the ability of ARTNet to accurately classify 44 reported bARTTs and negative samples. Then, exact domain subsequences were used to construct ARTNet, but overfitting occurred; therefore, we conducted a domain-based data optimization strategy and verified its effectiveness. Among the domain-based datasets, pos_art_346_random outperformed others due to its larger training sample size, underscoring the significance of large datasets in constructing DL models. Nevertheless, when the upstream and downstream information of the exact ART domain was completely destroyed rather than merely disrupted, pos_art_346_randomreplace did not exhibit any advantages. This suggests that the precise localization of ART-related domains may not be as accurate as anticipated, or alternatively, certain unidentified contextual features may play a crucial role in domain prediction. Besides, we empirically demonstrated the ability of ARTNet to predict novel bARTTs across domain superfamilies without sequence similarity. To optimize ARTNet, we also extensively explored alternative models, including three other DL models, and six well-established ML classifiers combined with 15 predefined features. Unsurprisingly, our CNN-based ARTNet outperformed the others in terms of both time efficiency and accuracy. Perhaps employing recently popular algorithms such as Transformer (Liu *et al.* 2024) instead of CNN to develop a bARTTs prediction model may potentially yield comparable or even superior results to our ARTNet. However, it may not significantly impact how we approach the scientific challenge of developing a new method for predicting bARTTs. To facilitate the future application of ARTNet, we have created a user-friendly online web server for the prediction of potential bARTTs. Nevertheless, comprehensive follow-up analyses of our predicted candidates are highly recommended to preclude potential false positives prior to further biological verification.

## 5 Conclusion

In this work, we developed a DL-based ARTNet for the prediction of ART toxins from bacterial genomes. We introduced an effective data augmentation method and a data optimization strategy to significantly enhance the performance of ARTNet. Our ARTNet achieved a Matthew’s correlation coefficient (MCC) of 0.9351 and an *F*1-score (macro) of 0.9666 on repeated independent test datasets, outperforming three other DL models and six traditional machine ML classifiers (combined with 15 predefined features) in terms of time efficiency and accuracy. In-depth analysis from multiple perspectives demonstrated the robustness and stability of ARTNet. Moreover, ARTNet has the potential to predict novel bARTTs across domain superfamilies without relying on sequence similarity. ARTNet trained on pos_art_346_random could provide more candidates and predict potential toxins belonging to other superfamily members that are very difficult to identify using sequence similarity-based methods. ARTNet trained on pos_art_346 and pos_whole may have higher specificity, as they performed strongly in identifying the 44 verified bARTTs. To offer more options and provide a more robust bARTT prediction service, we reported the results of ensemble ARTNet models trained on the three datasets described above on a user-friendly online web server. To the best of our knowledge, this is the first successful application of DL algorithms for the prediction of bARTTs. We anticipate that ARTNet will greatly facilitate the screening and identification of novel bARTTs from bacterial genomes by microbiologists. In addition, the ARTNet roadmap will benefit the development of future DL models for the identification of various bacterial virulence factors.

## Supplementary Material

btae378_Supplementary_Data

## Data Availability

The source codes and data are available at https://github.com/zhengdd0422/ARTNet/.
