# Supplementary material for: A deep learning method to predict bacterial ADP-ribosyltransferase toxins
Source: Bioinformatics. 2024 Jun 17;40(7):btae378. doi: 10.1093/bioinformatics/btae378 (PMC11219481; doi:10.1093/bioinformatics/btae378)
Supplement: btae378_Supplementary_Data [file btae378_supplementary_data.zip › Supplementary Methods.docx]

**Supplementary Methods**

**Traditional machine learning models using predefined features**

To verify the advantages of our proposed ARTNet over traditional ML methods using predefined features, we applied six well-established classification algorithms as the baselines([Xu, et al., 2021](#_ENREF_25); [Zeng and Zou, 2019](#_ENREF_27)).

**1. Feature engineering**

Building stable, dependable models with competitive performance requires thorough and efficient feature extraction([Xie, et al., 2021](#_ENREF_24)).To thoroughly study the typical and particular patterns of bARTTs proteins, we extracted a diverse array of features, which mainly fall into three major groups, including sequence-based features, physicochemical property-based features, and evolutionary information-based features.

**Group 1:** **sequence-based features group.** We extract three features including amino acid composition (AAC)([Anfinsen, 1972](#_ENREF_2)), dipeptide composition (DPC)([Zou, et al., 2013](#_ENREF_29)) and Tripeptide Compositions (TPC)([Chou, 2000](#_ENREF_5); [Hosen, et al., 2022](#_ENREF_11)) to describe the frequencies or compositions of sequence elements.

**(1) Amino Acids Composition**

AAC generates a 20-dimensional feature vector $\left\{ d1, d2, \ldots, d20 \right\}$for each sequence representing the frequency of each amino acid from the 20 standard amino acids in protein sequence. Each element in AAC vector is calculated as follows:

$d_{i}=\frac{c_{i}}{L}, for i=1,\ldots,20$,

where $c_{i}$is the number of occurrences of one of the 20 standard amino acids and $L$ is the length of the query sequence.

**(2) Dipeptide Composition**

DPC is encoded in a 400-dimensional feature vector $\left\{ f_{p1}, f_{p2}, \ldots, f_{p400} \right\}$ representing the frequency of each pair of amino acids from all $20 20$ possible pairs in the protein sequence. Each element $f_{pi}$ is calculated as follows:

$$f_{pi}=\frac{p_{i}}{L-1}, for i=1,\ldots,400,$$

where $p_{i}$is the number of appearances of the $i$-th amino acid pair and $L$ is the length of the query sequence.

**(3) Tripeptide Composition**

TPC is represented as an 8000-dimensional feature vector $\left\{ f_{p1}, f_{p2}, \ldots, f_{p8000} \right\}$, in which each feature represents the frequency of three adjacent amino acids from $20 20 20$ possible pairs in the protein sequence. Each $f_{pi}$ is calculated as follows:

$$f_{pi}=\frac{p_{i}}{L-2}, for i=1,\ldots,8000,$$

where $p_{i}$is the number of appearances of the $i$-th tripeptide and $L$ is the length of the query sequence.

**Group 2: Physicochemical property-based features group.** Physicochemical property-based features describe the statistical information about the physicochemical properties of amino acids in a protein sequence and have been widely used in many protein prediction tasks([Wang, et al., 2019](#_ENREF_19)). In this work, we extracted 4 features of this group: composition transition and distribution (CTD)([Cao, et al., 2013](#_ENREF_3)), quasi-sequence-order (QSO)([Chou, 2000](#_ENREF_5)), pseudo amino acid composition (PseAAC)([Chou, 2001](#_ENREF_6)) and autocorrelation features.

**(1) Composition, Transition and Distribution**

CTD refers to the composition, transition and distribution of protein sequences and represents the amino acid distribution pattern of a specific structural or physicochemical property along a protein. Seven types of physicochemical properties including hydrophobicity, charge, polarity, polarizability, normalized van der Waals volume, secondary structure, and solvent accessibility have been used for transforming the query protein sequence into 21 descriptors and 147 descriptor values([Cao, et al., 2013](#_ENREF_3)) .

**(2)** **Quasi-sequence-order**

QSO([Chou, 2000](#_ENREF_5)) describes the sequence order effect based on the physicochemical distance between 20 standard amino acids([Wang, et al., 2018](#_ENREF_21)). QSO is calculated as follows:

$$\tau_{d}=\sum_{i=1}^{L-d} {(d_{i, i+d})}^{2}, d=1, 2,\ldots,maxlag,$$

$$X_{r}= \frac{f_{r}}{\sum_{r=1}^{20} f_{r}+\omega\sum_{d=1}^{maxlag} \tau_{d}}, d=1, 2,\ldots, 20,$$

$X_{d}= \frac{{\omega\tau}_{d-20}}{\sum_{r=1}^{20} f_{r}+\omega\sum_{d=1}^{maxlag} \tau_{d}}, d=21, 22,\ldots, 20+maxlag$,

where $d_{i, i+d}$ is the distance between the two amino acids at position $i$and $i$+$d$, $L$ is the length of the query protein sequence, $maxlag$ is the maximum lag and $L$ must be not less than $maxlag$, $f_{r}$ represents the normalized occurrence for amino acid $i$, $\omega$ is a weighting factor. By setting $maxlag$ and $\omega$ to the default 30 and 0.1 respectively, propy([Cao, et al., 2013](#_ENREF_3)) uses the above formulas into Schneider-Wrede distance matrix([Schneider and Wrede, 1994](#_ENREF_17)) and Grantham distance matrix([Grantham, 1974](#_ENREF_9)) to derive two feature vectors, each of which combines $X_{r}$ and $X_{d}$ into 20+$maxlag$ dimensions, and finally obtains a 100-dimensional feature vector for QSO.

**(3)** **Pseudo-amino acid composition**

Since proposed by Kuo-Chen Chou in 2001, PseAAC has been widely used in the functional and structural analysis of proteins([Cao, et al., 2013](#_ENREF_3); [Zheng, et al., 2020](#_ENREF_28)). Instead of using the conventional 20-D amino acid composition to represent the sample of a protein, PseAAC merges sequence composition, physicochemical properties of amino acids including hydrophobicity, hydrophilicity and residue mass and sequence order information for improving protein subcellular localization and membrane protein type prediction. In this work, we applied propy to extract two types of PseAAC: type Ⅰ PseAAC (PAAC) and type Ⅱ PseAAC (APAAC). We set with a default value of 0.05, with a default value of 30 and get a 50-dimensional vector for each type of PseAAC.

**(4)** **autocorrelation features**

Autocorrelation features are defined based on the distribution of amino acid properties along the sequence and are used to describe the level of correlation between two protein or peptide sequences in terms of their specific structural or physicochemical property([Cao, et al., 2013](#_ENREF_3); [Li, et al., 2006](#_ENREF_14)). Eight amino acid properties including hydrophobicity, polarizability, free energy of amino acid solution, amino acid residue accessible surface areas, amino acid residue volumes, flexibility index of the B-factors of each amino acids, steric parameters derived from the van der Waals radius of amino acid side-chain atoms and relative mutability are used for deriving autocorrelation features. Each of these properties is centralized and standardized. In this work, we used propy to produce three autocorrelation features: normalized Moreau–Broto autocorrelation(MBauto)([Lin and Pan, 2001](#_ENREF_15)), Moran autocorrelation (Moranauto)([Horne, 1988](#_ENREF_10)) and Geary autocorrelation (Gearyauto)([Sokal and Thomson, 2006](#_ENREF_18)), each of which has descriptors and 240 descriptor values. They can be calculated as follows:

$$\mathrm{MBauto}\left( d \right)=\frac{\sum_{i=1}^{L-d} p_{i}p_{i+d}}{L-d}， d=1,2,\ldots,30,$$

$$\mathrm{Moranauto}\left( d \right)=\frac{\frac{1}{L-d}\sum_{i=1}^{L-d} (p_{i}-{\bar{p})(p}_{i+d}-\bar{p})}{\frac{1}{L}\sum_{i=1}^{L} {(p_{i}-\bar{p})}^{2}}， d=1,2,\ldots,30,$$

$$\mathrm{Gearyauto}\left( d \right)=\frac{\frac{1}{2(L-d)}\sum_{i=1}^{L-d} {(p_{i}-p_{i+d})}^{2}}{\frac{1}{L-1}\sum_{i=1}^{L} {(p_{i}-\bar{p})}^{2}}， d=1,2,\ldots,30,$$

where $L$ is the length of the protein sequence, $d$ is the lag of the autocorrelation, $p_{i}$ and  $p_{i+d}$ are the centralized and standardized amino acid property at position $i$  and $i$+$d$, $\bar{p}$ is the average of $p_{i}$.

**Group 3: evolutionary information-based features group.** An increasing number of researches have demonstrated that the study of protein sequences can benefit from the addition of evolutionary information such as position-specific scoring matrix (PSSM), which is more insightful than sequence information([An, et al., 2018](#_ENREF_1); [Wang, et al., 2019](#_ENREF_19); [Wang, et al., 2018](#_ENREF_21); [Wang, et al., 2011](#_ENREF_23)). For a protein sequence with a length of $L$, PSSM is a $L$×20 matrix, in which the $\left( i, j \right)$-th element denotes the probability of amino acid$j\left( j=1,2,\ldots,20 \right)$ to appear at the $i$-th position of the protein sequence([Wang, et al., 2019](#_ENREF_19)). We first applied PSI-BLAST search against the UniRef50 (accessed on May 2023) database with the parameters j = 3 and e-value = 0.001 to get the original PSSM profiles and then apply a python tool pssmpro based on POSSUM([Wang, et al., 2017](#_ENREF_22)) to generate the following PSSM profiles-based features:

**(1) PSSM-composition**

PSSM-composition represents a row transformation of the original PSSM by summing up all rows of the same amino acid residues([Zou, et al., 2013](#_ENREF_29)). Thus, a 20×20 matrix can be obtained using the following formula:

$$C_{i, j}=\frac{1}{L}\sum_{k=1}^{L} p_{k, j}\times{}_{k,j},$$

subject to:

$$\left\{ \begin{aligned} {}_{k,j}=1, r_{k}=a_{j} \\ {}_{k,j}=0, r_{k} a_{j} \end{aligned}, i, j=1,2,\ldots,20;k = 1,2,...,L, \right.$$

where $L$ is the length of the sequence, $r_{k}$ denotes the $k$th amino acid in query sequence, $a_{j}$ denotes the $j$th of 20 standard amino acids, $p_{k, j}$ denotes the element of the $k$th row and the $j$th column of the original PSSM.

**(2) S-FPSSM**

S-FPSSM is a row transformation based on a filtered matrix FPSSM**(**[**Zahiri, et al., 2013**](#_ENREF_26)**).** FPSSM is produced from the original PSSM in a preprocessing step during which all scores less than 0 are set to 0 and all scores greater than an expected value e to e (e=7). All elements in FPSSM are in the range of 0 to e, which avoids the influence of negative elements on positive elements when adding two elements during matrix transformation. Then a 20×20 matrix of S-FPSSM can be calculated by summing up all rows of the same amino acid based on the FPSSM, shown as follows:

$S_{i, j}=\sum_{k=1}^{L} {fp}_{k, j}\times{}_{k,j}$,

subject to:

$$\left\{ \begin{aligned} {}_{k,j}=1, r_{k}=a_{j} \\ {}_{k,j}=0, r_{k} a_{j} \end{aligned}, i, j=1,2,\ldots,20;k = 1,2,...,L, \right.$$

where $L$ is the length of the sequence, $r_{k}$ denotes the $k$th amino acid in the query sequence, $a_{j}$ denotes the $j$th of 20 standard amino acids, ${fp}_{k, j}$ denotes the element of the $k$th row and the $j$th column of FPSSM.

**(3) DPC-PSSM**

DPC-PSSM is a column transformation of the original PSSM profiles that integrates sequence-order information DPC into evolutionary information([Liu, et al., 2010](#_ENREF_16)). It is a 400-dimentional feature vector that can be calculated as follows:

$$d_{i,j}=\frac{1}{L-1}\sum_{k=1}^{L-1} p_{k, i}\times p_{k+1,j}, i, j=1,2,\ldots,20,$$

where $L$ is the length of the sequence as well as the total row counts of original PSSM, $p_{k, i}$ is the element at $k$th row and $i$th column of the original PSSM.

**(4)** **Pse-PSSM**

Pse-PSSM is a mixture of row and column transformations and is combination of the mean of 20 columns and the correlation factors correspond to 20 columns in PSSM Matrix([Chou and Shen, 2007](#_ENREF_7)). Based on the PSSM transformation and dimension normalization of the resulting feature vector, Pse-PSSM is a dependable feature encoding technique for obtaining evolutionary information([Wang, et al., 2018](#_ENREF_21)). It can be calculated as follows:

$$T_{i,j}=\frac{E_{i, j}-\frac{\sum_{k=1}^{20} E_{i,k}}{20}}{\sqrt{\frac{\sum_{k=1}^{20} (E_{i,k}-\frac{\sum_{k=1}^{20} E_{i,k}}{20})}{20}}}, i=1,2,\ldots,L,$$

$H_{j}^{\alpha}=\frac{1}{L-\alpha}\sum_{i=1}^{L-\alpha} {(T_{i,j}-T_{i+\alpha,j})}^{2}$*，*

$\bar{T_{j}}=\frac{1}{L}\sum_{i=1}^{L} T_{i,j}$*,*

$T^{'}=[\bar{T_{1}}, \bar{T_{2}}, \ldots, \bar{T_{20}}]$*,*

$H^{'}=[H_{1}^{\alpha}, H_{2}^{\alpha}, \ldots, H_{20}^{\alpha}]$*,*

$P={[T}^{'},H^{'}]$,

where L is the length of the protein sequence, $E_{i,k}$ denotes the element in the $i$th row and $k$th column of the original PSSM. We use $\alpha$ with a default value of 1 in pssmpro and get a 40-dimentional feature vector.

**(5)** **RPSSM**

RPSSM is created by combining PSSM based on amino acid similarity and then transforming the features with the autocovariance([Chen, et al., 2023](#_ENREF_4)). The original PSSM of $L\times$20 is first simplified to $L\times$10 matrix in accordance with the amino acid similarity criteria proposed by Li et al.([Li, et al., 2003](#_ENREF_13)), and the amino acid pseudo-composition is extracted from the simplified PSSM. Protein sequence dipeptide composition is then applied to produce RPSSM to account for local sequence order effects. Finally, a 110-dimensional vector is extracted from each query protein sequence. More information can be found in Stack-VTP([Chen, et al., 2023](#_ENREF_4)).

Feature selection is the selection of features that are most useful and relevant to the underlying problems. In this work, we used SelectFromModel (estimator=LogisticRegression, max_features=100), a meta-transformer based on importance weights, in scikit-learn to perform the feature selection on TPC to reduce its high dimensionality before modeling. To avoid the prediction results on a specific feature being dominated by its larger numerical values, we employed Min-Max scaling([Kahng, et al., 2002](#_ENREF_12)) to normalize values of the predefined features so that all values fall into the same numerical interval from 0 to 1. Min-Max scaling is calculated as following formula:

$$x^{'}=\frac{x-x_{min}}{x_{max}-x_{min}},$$

where $x$ is an original value, $x^{'}$ is the normalized value, $x_{min}$ and $x_{max}$ describe the minimum value and the maximum value in the feature vector, respectively.

**2. Traditional machine learning classifiers**

Based on the predefined features, we applied six well-established popular supervised ML algorithms widely used in protein predicting tasks and implement them with python packages from scikit-learn with default parameters to construct ART predicting methods. LR is a mainly used for classification tasks where the goal is to predict the probability that an instance of belonging to a given class or not by analyzing the relationship between a set of independent variables and the dependent binary variables([Wang, et al., 2017](#_ENREF_20)). SVM is one of the most robust prediction methods, being based on statistical learning frameworks or Vapnik–Chervonenkis theory. RF classifier is an ensemble method that trains several decision trees in parallel with bootstrapping followed by aggregation, jointly referred as bagging([Xie, et al., 2021](#_ENREF_24)). KNN is a non-parametric, powerful classification method, which predicts a new candidate by evaluating the distance functions to k nearest known neighbors([Wang, et al., 2017](#_ENREF_20)). GBC gives a prediction model in the form of an ensemble of weak prediction models such as decision trees and report the final class with the majority of votes from weak learners([Goodswen, et al., 2021](#_ENREF_8)). XGBC is an effective implementation and extension of gradient boosting decision tree that can generate a strong learning model by linearly integrating weak learning models composed of decision trees([Xie, et al., 2021](#_ENREF_24)).

**References**

An, Y.*, et al.* (2018) Comprehensive assessment and performance improvement of effector protein predictors for bacterial secretion systems III, IV and VI, *Briefings in bioinformatics*, **19**, 148-161.

Anfinsen, C.B. (1972) The formation and stabilization of protein structure, *The Biochemical journal*, **128**, 737-749.

Cao, D.S., Xu, Q.S. and Liang, Y.Z. (2013) propy: a tool to generate various modes of Chou's PseAAC, *Bioinformatics*, **29**, 960-962.

Chen, Y., Gao, L. and Zhang, T. (2023) Stack-VTP: prediction of vesicle transport proteins based on stacked ensemble classifier and evolutionary information, *BMC Bioinformatics*, **24**, 137.

Chou, K.C. (2000) Prediction of protein subcellular locations by incorporating quasi-sequence-order effect, *Biochemical and biophysical research communications*, **278**, 477-483.

Chou, K.C. (2001) Prediction of protein cellular attributes using pseudo-amino acid composition, *Proteins*, **43**, 246-255.

Chou, K.C. and Shen, H.B. (2007) MemType-2L: a web server for predicting membrane proteins and their types by incorporating evolution information through Pse-PSSM, *Biochemical and biophysical research communications*, **360**, 339-345.

Goodswen, S.J.*, et al.* (2021) Machine learning and applications in microbiology, *FEMS microbiology reviews*, **45**.

Grantham, R. (1974) Amino acid difference formula to help explain protein evolution, *Science*, **185**, 862-864.

Horne, D.S. (1988) Prediction of protein helix content from an autocorrelation analysis of sequence hydrophobicities, *Biopolymers*, **27**, 451-477.

Hosen, M.F.*, et al.* (2022) DeepDNAbP: A deep learning-based hybrid approach to improve the identification of deoxyribonucleic acid-binding proteins, *Computers in biology and medicine*, **145**, 105433.

Kahng, A.B., Mantik, S. and Markov, I.L. (2002) Min-max placement for large-scale timing optimization. *Proceedings of the 2002 international symposium on Physical design*. pp. 143-148.

Li, T.*, et al.* (2003) Reduction of protein sequence complexity by residue grouping, *Protein engineering*, **16**, 323-330.

Li, Z.R.*, et al.* (2006) PROFEAT: a web server for computing structural and physicochemical features of proteins and peptides from amino acid sequence, *Nucleic acids research*, **34**, W32-37.

Lin, Z. and Pan, X.M. (2001) Accurate prediction of protein secondary structural content, *Journal of protein chemistry*, **20**, 217-220.

Liu, T., Zheng, X. and Wang, J. (2010) Prediction of protein structural class for low-similarity sequences using support vector machine and PSI-BLAST profile, *Biochimie*, **92**, 1330-1334.

Schneider, G. and Wrede, P. (1994) The rational design of amino acid sequences by artificial neural networks and simulated molecular evolution: de novo design of an idealized leader peptidase cleavage site, *Biophysical journal*, **66**, 335-344.

Sokal, R.R. and Thomson, B.A. (2006) Population structure inferred by local spatial autocorrelation: an example from an Amerindian tribal population, *American journal of physical anthropology*, **129**, 121-131.

Wang, J.*, et al.* (2019) Bastion3: a two-layer ensemble predictor of type III secreted effectors, *Bioinformatics*, **35**, 2017-2028.

Wang, J.*, et al.* (2017) Systematic analysis and prediction of type IV secreted effector proteins by machine learning approaches, *Briefings in bioinformatics*.

Wang, J.*, et al.* (2018) Bastion6: a bioinformatics approach for accurate prediction of type VI secreted effectors, *Bioinformatics*, **34**, 2546-2555.

Wang, J.*, et al.* (2017) POSSUM: a bioinformatics toolkit for generating numerical sequence feature descriptors based on PSSM profiles, *Bioinformatics*, **33**, 2756-2758.

Wang, Y.*, et al.* (2011) High-accuracy prediction of bacterial type III secreted effectors based on position-specific amino acid composition profiles, *Bioinformatics*, **27**, 777-784.

Xie, R.*, et al.* (2021) DeepVF: a deep learning-based hybrid framework for identifying virulence factors using the stacking strategy, *Briefings in bioinformatics*, **22**, 1-15.

Xu, J.*, et al.* (2021) Comprehensive assessment of machine learning-based methods for predicting antimicrobial peptides, *Briefings in bioinformatics*, **22**.

Zahiri, J.*, et al.* (2013) PPIevo: protein-protein interaction prediction from PSSM based evolutionary information, *Genomics*, **102**, 237-242.

Zeng, C. and Zou, L. (2019) An account of in silico identification tools of secreted effector proteins in bacteria and future challenges, *Briefings in bioinformatics*, **20**, 110-129.

Zheng, D.*, et al.* (2020) Learning transferable deep convolutional neural networks for the classification of bacterial virulence factors, *Bioinformatics*, **36**, 3693-3702.

Zou, L., Nan, C. and Hu, F. (2013) Accurate prediction of bacterial type IV secreted effectors using amino acid composition and PSSM profiles, *Bioinformatics*, **29**, 3135-3142.
