# Supplementary material for: A deep learning method to predict bacterial ADP-ribosyltransferase toxins
Source: Bioinformatics. 2024 Jun 17;40(7):btae378. doi: 10.1093/bioinformatics/btae378 (PMC11219481; doi:10.1093/bioinformatics/btae378)
Supplement: btae378_Supplementary_Data [file btae378_supplementary_data.zip › Supplementary Figures and Tables.docx]

**Supplementary Figures and Tables**


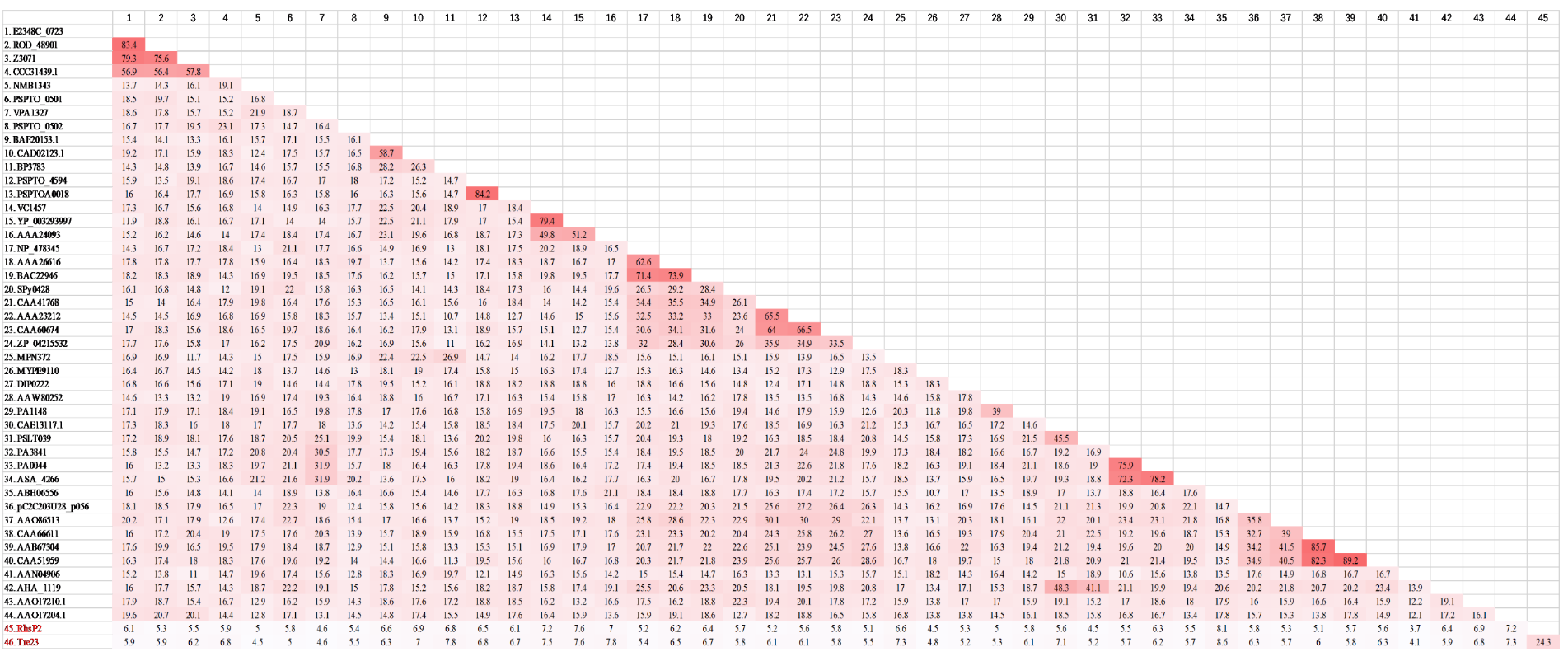


**Figure S1**. The identity of the global sequence alignment of Rhsp2, Tre23 and 44 experimentally verified bARTTs (ART domain). Rhsp2 and Tre23 are highlighted in red.

**
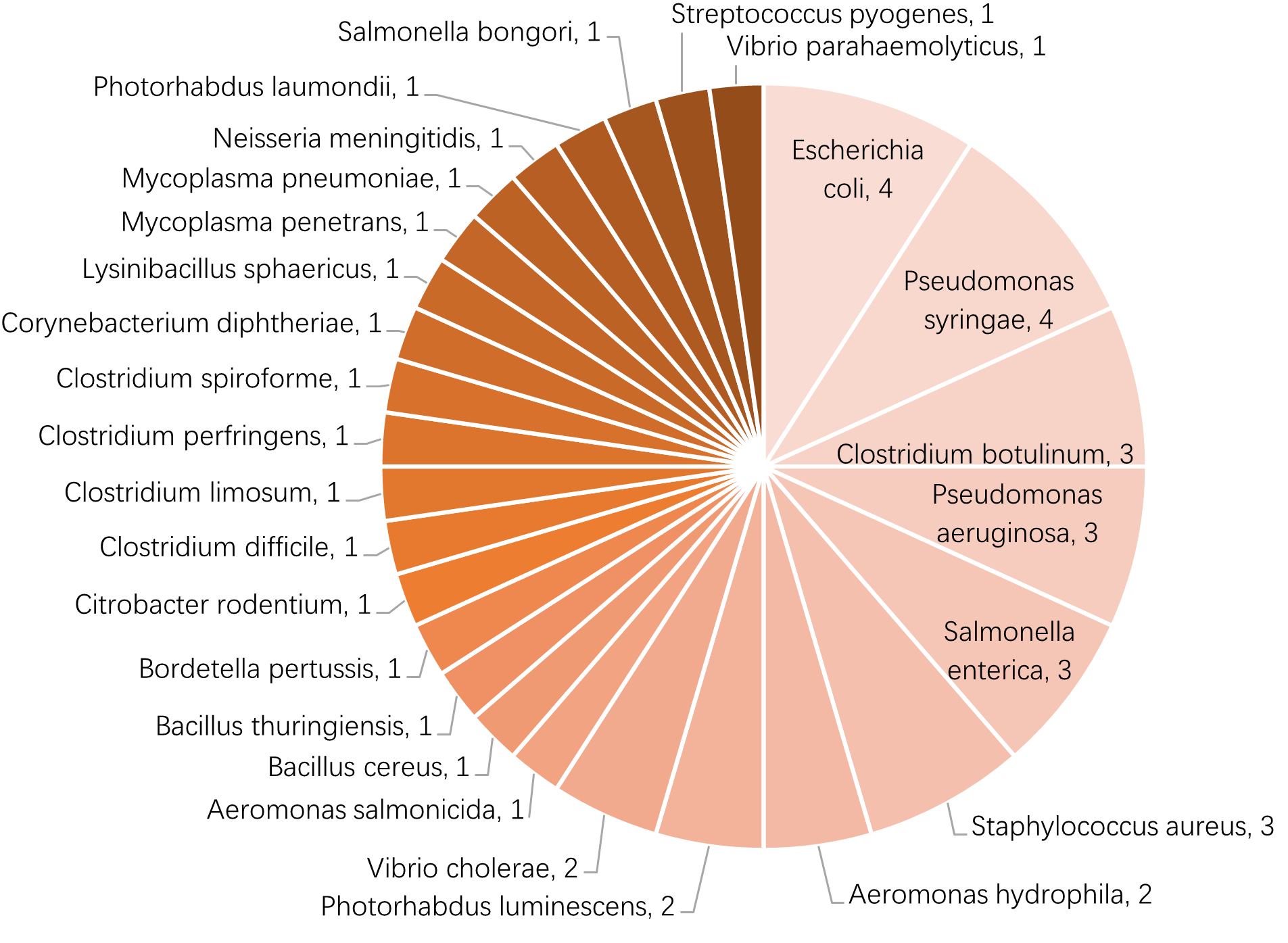
**

**Figure S2**. The distribution of 44 experimentally verified bacterial ADP-ribosyltransferase toxins in bacterial genomes.


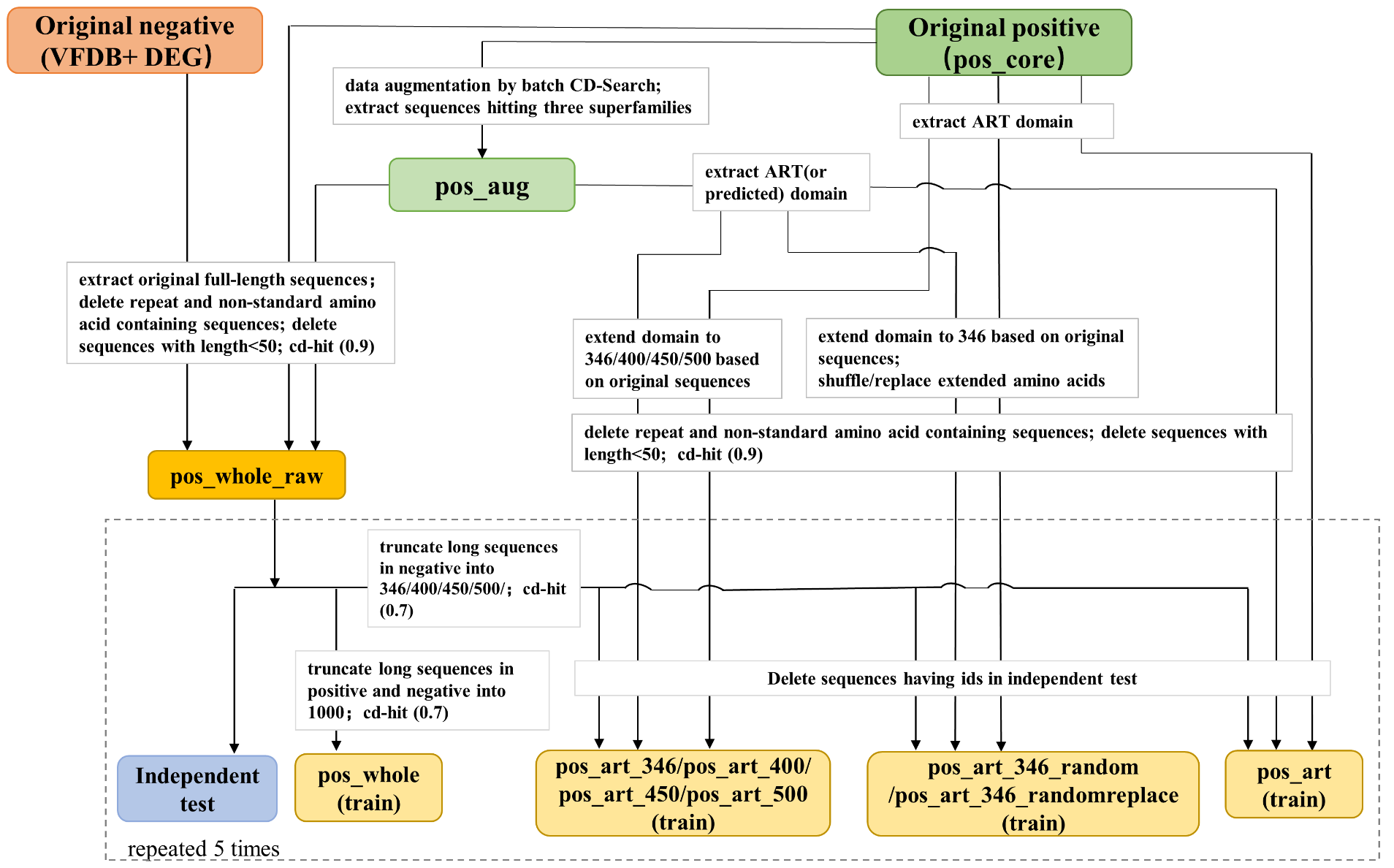


**Figure S3**. Flow diagram of data preprocessing. The pos_core refers to 44 experimentally verified bARTTs. The pos_aug represents raw samples after data augmentation. The pos_whole_raw denotes full-length sequences after preprocessing based on data augmentation. The pos_art(train), pos_art_346(train), pos_art_400(train), pos_art_450(train), pos_art_500(train), pos_art_346_random(train), pos_art_346_randomreplace(train), and pos_whole(train) refer to the final train samples of each dataset after data preprocessing.


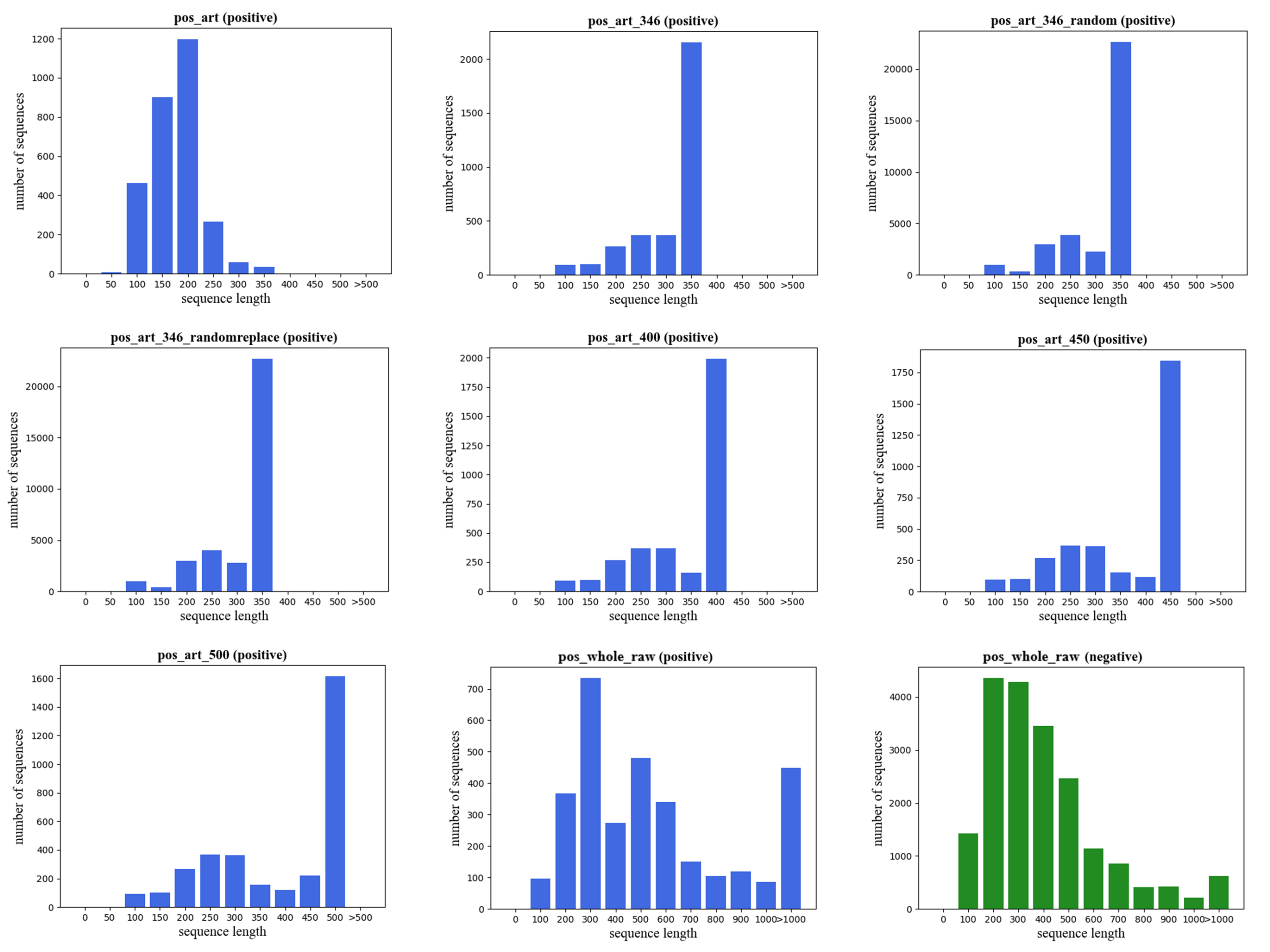


**Figure S4**. Length distribution of 8 datasets after data preprocessing. Positive sample sets are marked in blue, and the original full-length negative sample set is marked in green.

**
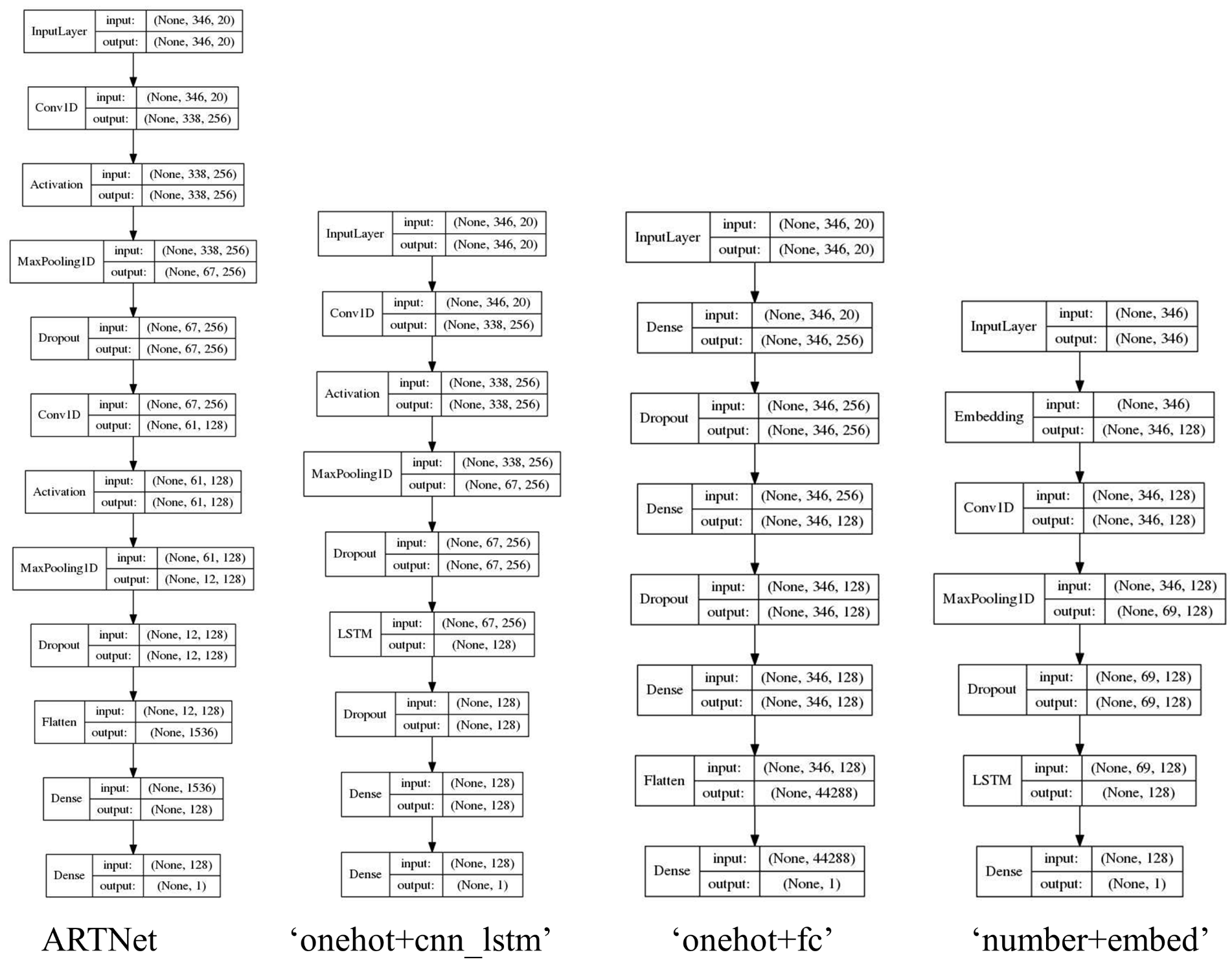
Figure S5.** Model structure details of four deep learning models.


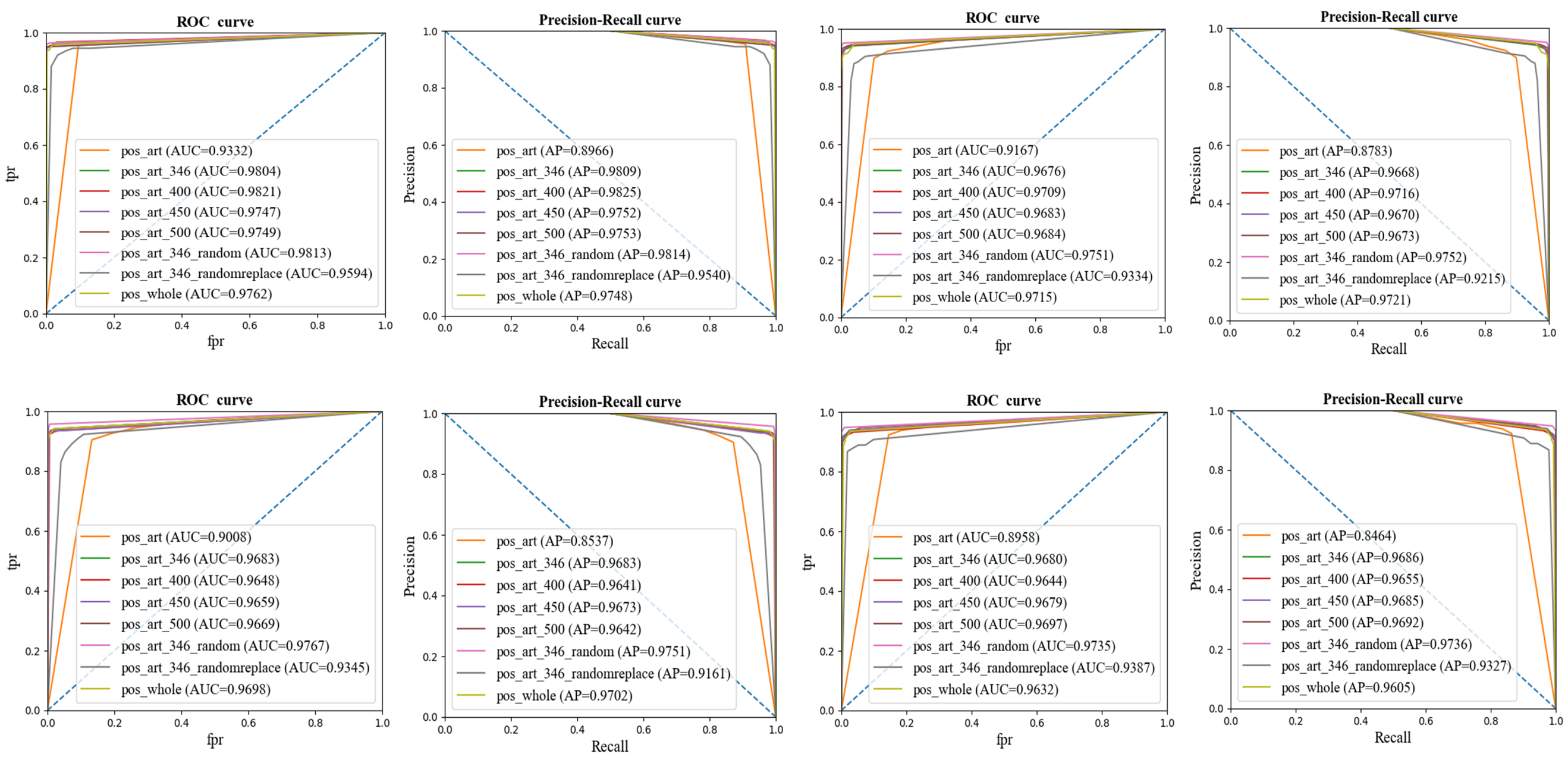


**Figure S6**. ROC curves and precision-recall curves of ARTNet on the other 4 of 5 repeated independent test datasets. Each figure represents the results of one independent test dataset. Only the results of the best length threshold of each model are plotted.


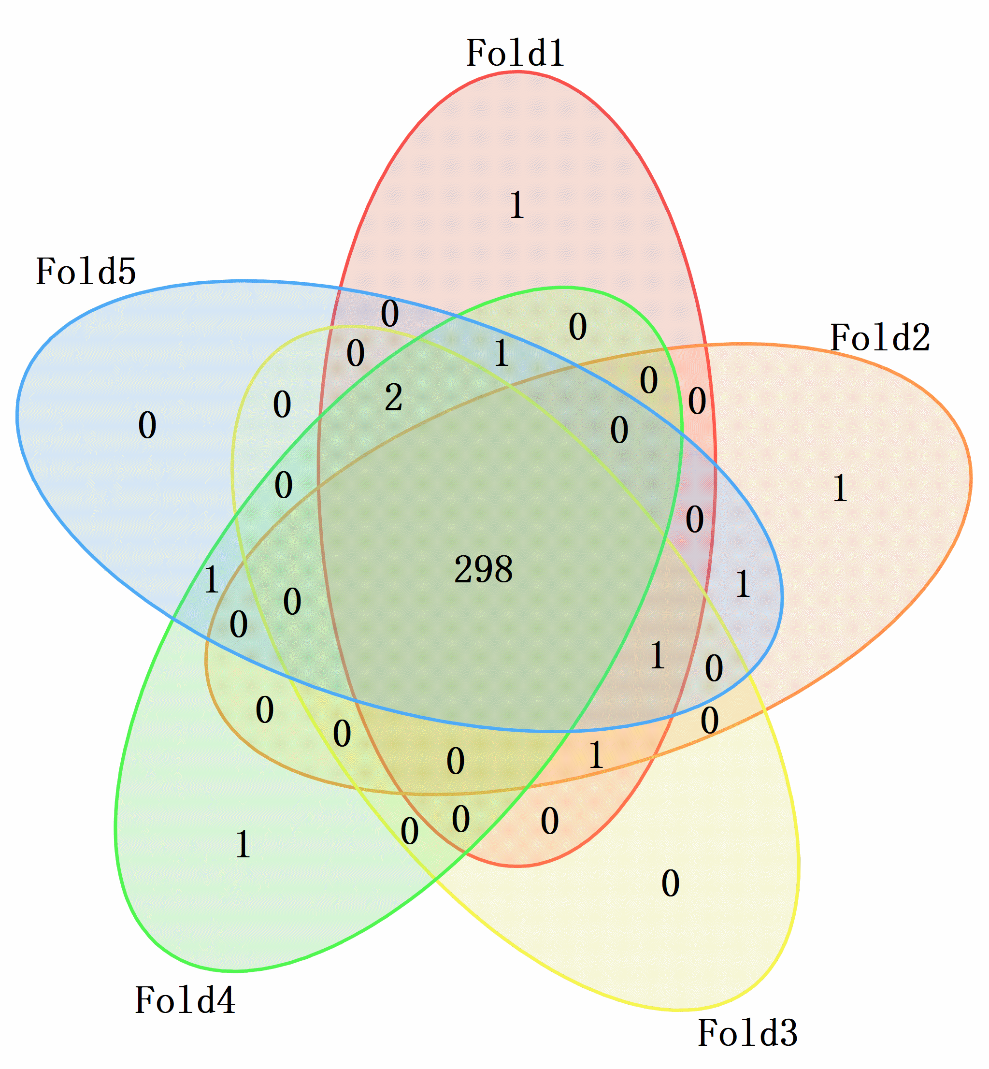


**Figure S7.** Venn diagram of 5 ARTNet models trained on pos_art_346_random for predicting true positives in one independent test.


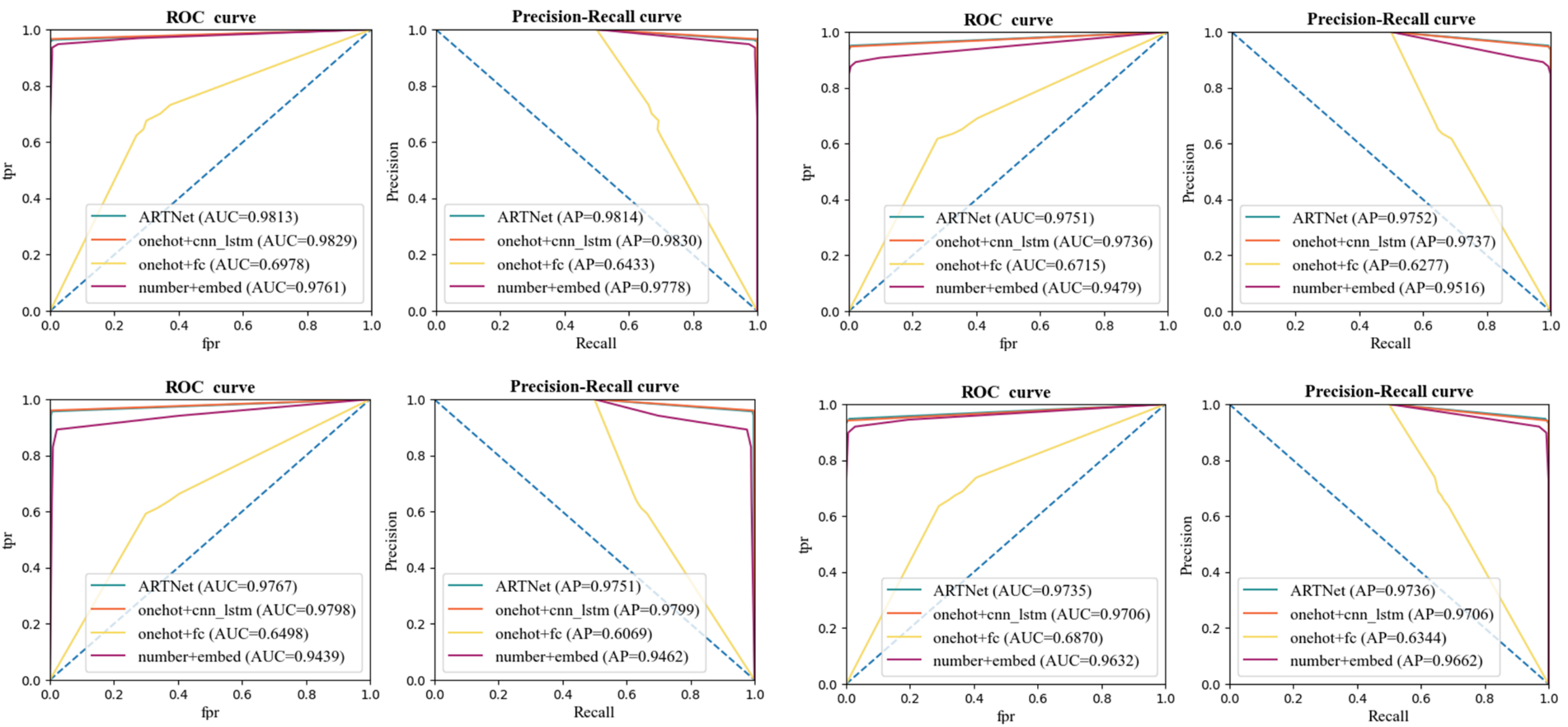


**Figure S8.** ROC curves and precision-recall curves of four deep learning models combined with pos_art_346_random on the other 4 of 5 repeated independent tests. Each figure represents the results of one independent test dataset.


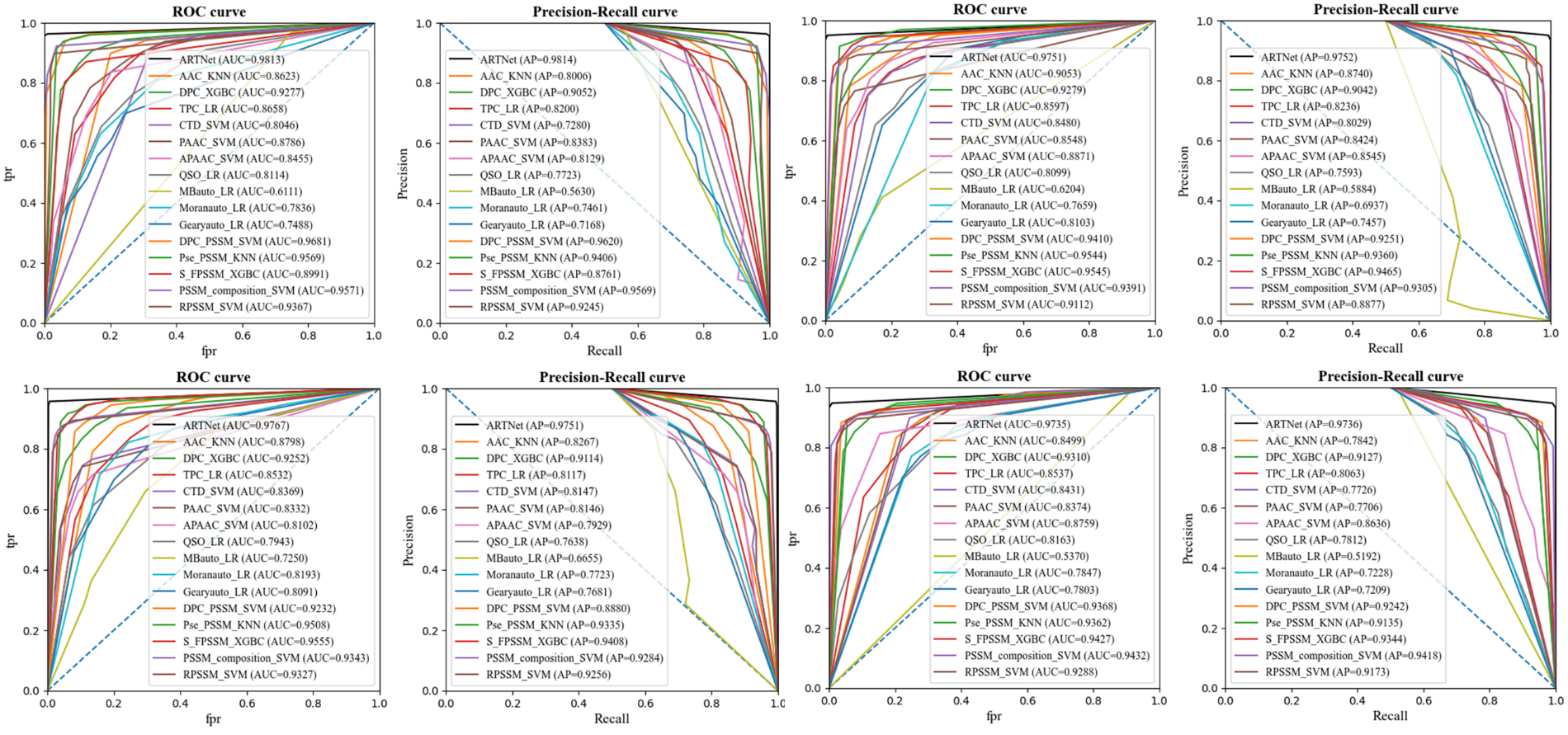


**Figure S9**. ROC curves and precision-recall curves of ARTNet combined with pos_art_346_random and 6 traditional machine learning models combined with 15 predefined features on the other 4 of 5 repeated independent tests. On each independent test, only the best model for each feature is plotted.

**Table S2.** Basic statistics of 8 datasets in 1 of 5 repeated experiments.

| **Method\Samples number** | **Tain** | | | **Independent test** | | |
| --- | --- | --- | --- | --- | --- | --- |
|  | **pos_core** | **pos_aug** | **negative** | **pos_core** | **pos_aug** | **negative** |
| pos_whole | 34 | 5051 | 20062 | 10 | 314 | 324 |
| pos_art | 34 | 2645 | 33299 |  |  |  |
| pos_art_346 | 34 | 3012 | 33293 |  |  |  |
| pos_art_400 | 34 | 3002 | 28915 |  |  |  |
| pos_art_450 | 34 | 2965 | 26383 |  |  |  |
| pos_art_500 | 34 | 2954 | 24670 |  |  |  |
| pos_art_346_random | 34 | 32662 | 33299 |  |  |  |
| pos_art_346_randomreplace | 34 | 22408 | 33299 |  |  |  |

*Note*: pos_core refers to 44 experimentally verified bARTTs, pos_aug represents samples derived from data augmentation.

**Table S4.** Performance (mean+/-standard deviation) of ARTNet combined with pos_art_346_random using different negative datasets on 5-fold cross-validation.

| **Data** | **Accuracy** | **Sensitivity** | **Specificity** | **F1_score(micro)** | **Precision(macro)** | **Recall(macro)** | **F1_score(macro)** | **MCC** |
| --- | --- | --- | --- | --- | --- | --- | --- | --- |
| VFDB | 0.9786(+/-0.0046) | **0.9407(+/-0.0076)** | 0.9886(+/-0.0062) | **0.9398(+/-0.0139)** | 0.9894(+/-0.0086) | 0.9790(+/-0.0047) | 0.9790(+/-0.0047) | 0.9790(+/-0.0047) |
| DEG | 0.9868(+/-0.0023) | 0.9314(+/-0.0062) | 0.9950(+/-0.0025) | 0.9306(+/-0.0043) | 0.9953(+/-0.0017) | 0.9869(+/-0.0017) | 0.9869(+/-0.0017) | 0.9869(+/-0.0017) |
| VFDB+DEG | **0.9896(+/-0.0038)** | 0.9261(+/-0.0098) | **0.9957(+/-0.0045)** | 0.9285(+/-0.0114) | **0.9967(+/-0.0013)** | **0.9907(+/-0.0012)** | **0.9907(+/-0.0012)** | **0.9907(+/-0.0012)** |

*Note*: The best indicators are shown in bold.

**Table S5.** Performance comparison (mean+/-standard deviation) of four deep learning models combined with pos_art_346_random on repeated 5-fold cross-validation.

| **Model** | **Accuracy** | **Sensitivity** | **Specificity** | **F1_score(micro)** | **Precision(macro)** | **Recall(macro)** | **F1_score(macro)** | **MCC** |
| --- | --- | --- | --- | --- | --- | --- | --- | --- |
| ARTNet | **0.9956(+/-0.0002)** | **0.9922(+/-0.0003)** | 0.9990(+/-0.0001) | **0.9956(+/-0.0002)** | **0.9957(+/-0.0002)** | **0.9956(+/-0.0002)** | **0.9956(+/-0.0002)** | **0.9912(+/-0.0004)** |
| onehot+cnn_lstm | 0.9952(+/-0.0002) | 0.9913(+/-0.0005) | **0.9991(+/-0.0001)** | 0.9952(+/-0.0002) | 0.9953(+/-0.0002) | 0.9952(+/-0.0002) | 0.9952(+/-0.0002) | 0.9904(+/-0.0005) |
| onehot+fc | 0.9467(+/-0.0012) | 0.9391(+/-0.0026) | 0.9541(+/-0.0011) | 0.9467(+/-0.0012) | 0.9469(+/-0.0011) | 0.9466(+/-0.0012) | 0.9467(+/-0.0012) | 0.8936(+/-0.0023) |
| number+embed | 0.9060(+/-0.0601) | 0.9146(+/-0.1038) | 0.8976(+/-0.1000) | 0.9060(+/-0.0601) | 0.8768(+/-0.0866) | 0.9061(+/-0.0601) | 0.8861(+/-0.0775) | 0.8130(+/-0.1204) |

*Note*: The best indicators are shown in bold.

**Table S6.** Performance comparison (mean+/-standard deviation) of four deep learning models trained on pos_art_346_random on repeated independent test datasets.

| **Model** | **Accuracy** | **Sensitivity** | **Specificity** | **F1_score(micro)** | **Precision(macro)** | **Recall(macro)** | **F1_score(macro)** | **MCC** |
| --- | --- | --- | --- | --- | --- | --- | --- | --- |
| ARTNet | 0.9666(+/-0.0058) | 0.9354(+/-0.0116) | 0.9978(+/-0.0009) | 0.9666(+/-0.0058) | 0.9685(+/-0.0052) | 0.9666(+/-0.0058) | 0.9666(+/-0.0058) | 0.9351(+/-0.0110) |
| onehot+cnn_lstm | **0.9678(+/-0.0069)** | **0.9369(+/-0.0146)** | **0.9988(+/-0.0008)** | **0.9678(+/-0.0069)** | **0.9698(+/-0.0062)** | **0.9678(+/-0.0069)** | **0.9678(+/-0.0070)** | **0.9376(+/-0.0131)** |
| onehot+fc | 0.6565(+/-0.0172) | 0.6542(+/-0.0228) | 0.6588(+/-0.0151) | 0.6565(+/-0.0172) | 0.6567(+/-0.0173) | 0.6565(+/-0.0172) | 0.6564(+/-0.0172) | 0.3131(+/-0.0346) |
| number+embed | 0.8563(+/-0.0587) | 0.8409(+/-0.1015) | 0.8717(+/-0.0984) | 0.8563(+/-0.0587) | 0.8303(+/-0.0847) | 0.8563(+/-0.0587) | 0.8360(+/-0.0763) | 0.7165(+/-0.1169) |

*Note*: The best indicators are shown in bold.

**Table S7.** Performance comparison (mean+/-standard deviation) of four deep learning models combined with pos_art_346_random on 5-fold cross-validation to predict proteins across superfamilies.

| **Model** | **Accuracy** | **Sensitivity** | **Specificity** | **F1_score(micro)** | **Precision(macro)** | **Recall(macro)** | **F1_score(macro)** | **MCC** |
| --- | --- | --- | --- | --- | --- | --- | --- | --- |
| ARTNet | **0.9962(+/-0.0002)** | **0.9930(+/-0.0003)** | **0.9993(+/-0.0002)** | **0.9962(+/-0.0002)** | **0.9962(+/-0.0002)** | **0.9961(+/-0.0002)** | **0.9962(+/-0.0002)** | **0.9923(+/-0.0005)** |
| onehot+cnn_lstm | 0.9957(+/-0.0003) | 0.9921(+/-0.0005) | 0.9993(+/-0.0002) | 0.9957(+/-0.0003) | 0.9958(+/-0.0003) | 0.9957(+/-0.0003) | 0.9957(+/-0.0003) | 0.9915(+/-0.0006) |
| onehot+fc | 0.9469(+/-0.0025) | 0.9373(+/-0.0105) | 0.9564(+/-0.0073) | 0.9469(+/-0.0025) | 0.9473(+/-0.0022) | 0.9469(+/-0.0025) | 0.9469(+/-0.0025) | 0.8941(+/-0.0047) |
| number+embed | 0.9803(+/-0.0133) | 0.9720(+/-0.0155) | 0.9885(+/-0.0193) | 0.9803(+/-0.0133) | 0.9807(+/-0.0133) | 0.9802(+/-0.0133) | 0.9803(+/-0.0133) | 0.9610(+/-0.0266) |

*Note*: The best indicators are shown in bold.

**Table S8.** Performance comparison (mean+/-standard deviation) of four deep learning models trained on pos_art_346_random on an independent test dataset to predict proteins across superfamilies.

| **Model** | **Accuracy** | **Sensitivity** | **Specificity** | **F1_score(micro)** | **Precision(macro)** | **Recall(macro)** | **F1_score(macro)** | **MCC** |
| --- | --- | --- | --- | --- | --- | --- | --- | --- |
| ARTNet | **0.9031(+/-0.0021)** | **0.8073(+/-0.0040)** | **0.9989(+/-0.0015)** | **0.9031(+/-0.0021)** | **0.9184(+/-0.0017)** | **0.9031(+/-0.0021)** | **0.9022(+/-0.0022)** | **0.8214(+/-0.0038)** |
| onehot+cnn_lstm | 0.8908(+/-0.0088) | 0.7838(+/-0.0187) | 0.9978(+/-0.0012) | 0.8908(+/-0.0088) | 0.9096(+/-0.0055) | 0.8908(+/-0.0088) | 0.8895(+/-0.0092) | 0.8002(+/-0.0144) |
| onehot+fc | 0.6296(+/-0.0082) | 0.5743(+/-0.0246) | 0.6849(+/-0.0188) | 0.6296(+/-0.0082) | 0.6314(+/-0.0078) | 0.6296(+/-0.0082) | 0.6283(+/-0.0086) | 0.2610(+/-0.0159) |
| number+embed | 0.7581(+/-0.0760) | 0.5441(+/-0.1521) | 0.9721(+/-0.0366) | 0.7581(+/-0.0760) | 0.8206(+/-0.0462) | 0.7581(+/-0.0760) | 0.7421(+/-0.0933) | 0.5727(+/-0.1243) |

*Note*: The best indicators are shown in bold.

**Table S9.** Performance comparison (mean+/-standard deviation) of ARTNet combined with pos_art_346 and pos_whole on 5-fold cross validation to predict proteins across superfamilies.

| **Data** | **Accuracy** | **Sensitivity** | **Specificity** | **F1_score(micro)** | **Precision(macro)** | **Recall(macro)** | **F1_score(macro)** | **MCC** |
| --- | --- | --- | --- | --- | --- | --- | --- | --- |
| pos_art_346 | 0.9930(+/-0.0019) | 0.9468(+/-0.0060) | 0.9972(+/-0.0022) | 0.9930(+/-0.0019) | 0.9817(+/-0.0116) | 0.9720(+/-0.0029) | 0.9767(+/-0.0062) | 0.9536(+/-0.0124) |
| pos_whole | 0.9879(+/-0.0014) | 0.9638(+/-0.0053) | 0.9942(+/-0.0022) | 0.9879(+/-0.0014) | 0.9840(+/-0.0039) | 0.9790(+/-0.0022) | 0.9814(+/-0.0021) | 0.9629(+/-0.0043) |

**Table S10.** Performance comparison (mean+/-standard deviation) of ARTNet trained on pos_art_346 and pos_whole on an independent test dataset to predict proteins across superfamilies.

| **Data** | **Accuracy** | **Sensitivity** | **Specificity** | **F1_score(micro)** | **Precision(macro)** | **Recall(macro)** | **F1_score(macro)** | **MCC** |
| --- | --- | --- | --- | --- | --- | --- | --- | --- |
| pos_art_346 | 0.6721(+/-0.0092) | 0.3514(+/-0.0195) | 0.9927(+/-0.0042) | 0.6721(+/-0.0092) | 0.7925(+/-0.0061) | 0.6721(+/-0.0092) | 0.6344(+/-0.0130) | 0.4485(+/-0.0150) |
| pos_whole | 0.5243(+/-0.0108) | 0.0570(+/-0.0250) | 0.9916(+/-0.0071) | 0.5243(+/-0.0108) | 0.6955(+/-0.0314) | 0.5243(+/-0.0108) | 0.3909(+/-0.0240) | 0.1350(+/-0.0360) |
