# Supplementary figures and images for: A deep learning method to predict bacterial ADP-ribosyltransferase toxins
Source: Bioinformatics. 2024 Jun 17;40(7):btae378. doi: 10.1093/bioinformatics/btae378 (PMC11219481; doi:10.1093/bioinformatics/btae378)

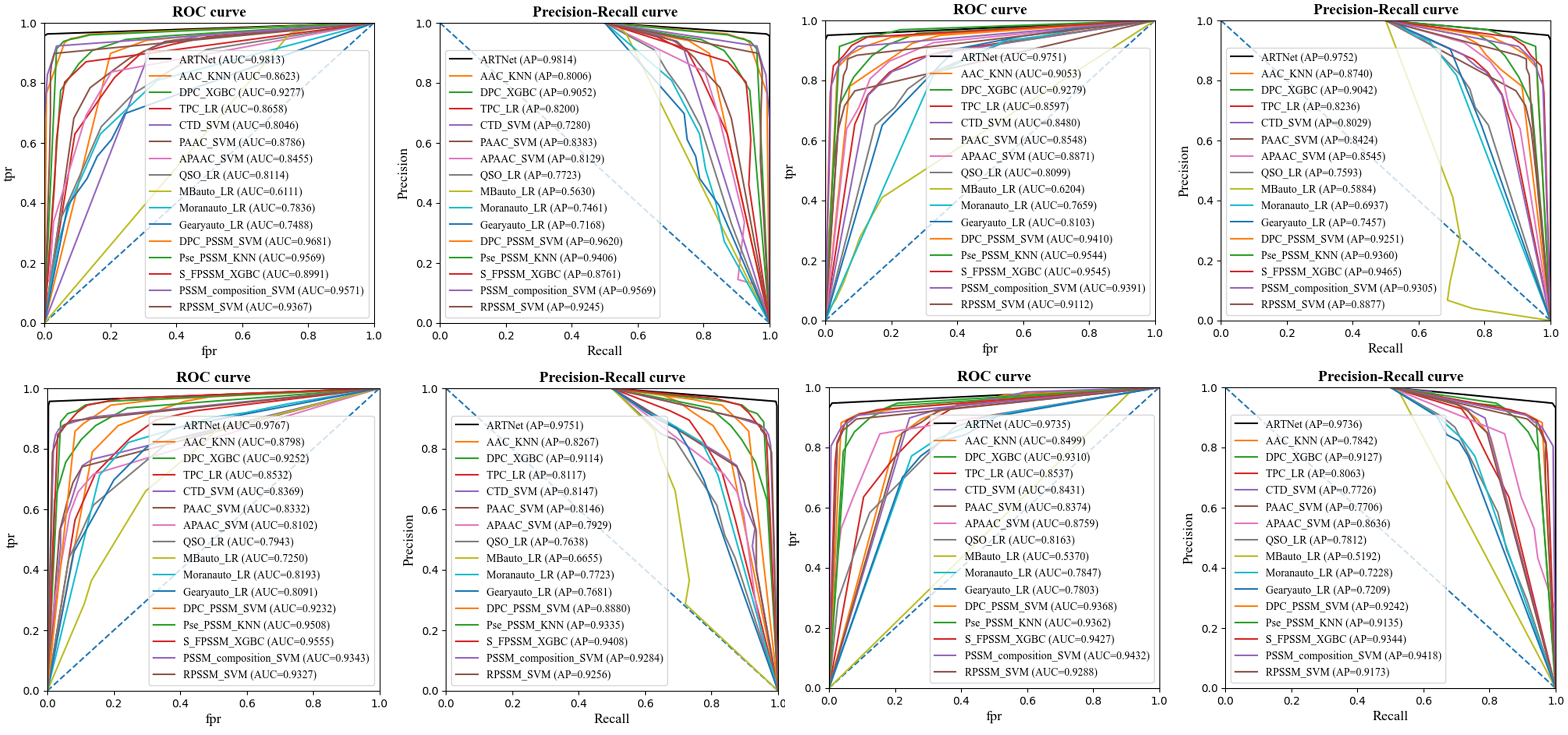

Supplement: btae378_Supplementary_Data [file btae378_supplementary_data.zip › Figure S9.tif]

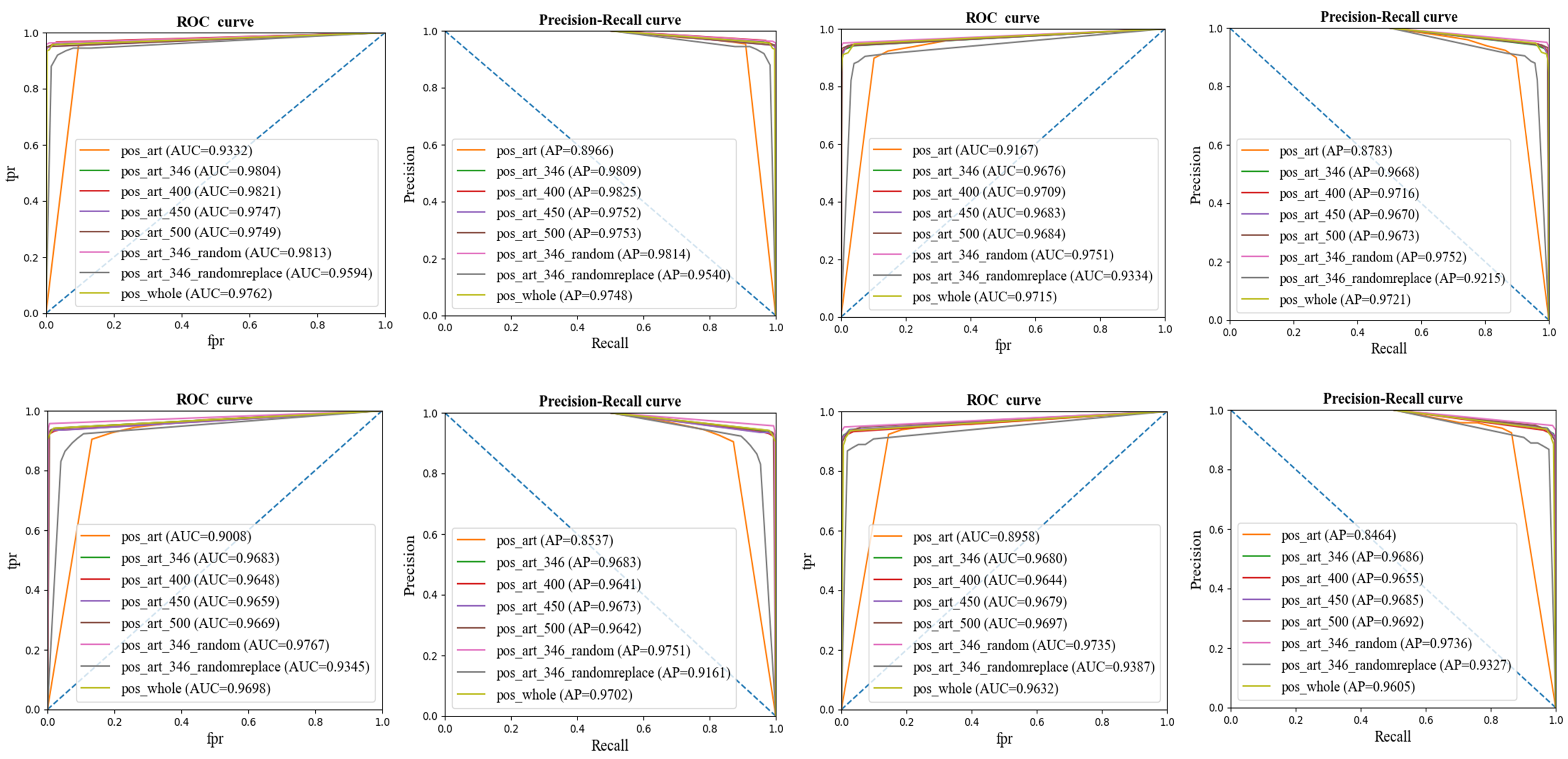

Supplement: btae378_Supplementary_Data [file btae378_supplementary_data.zip › Figure S6.tif]

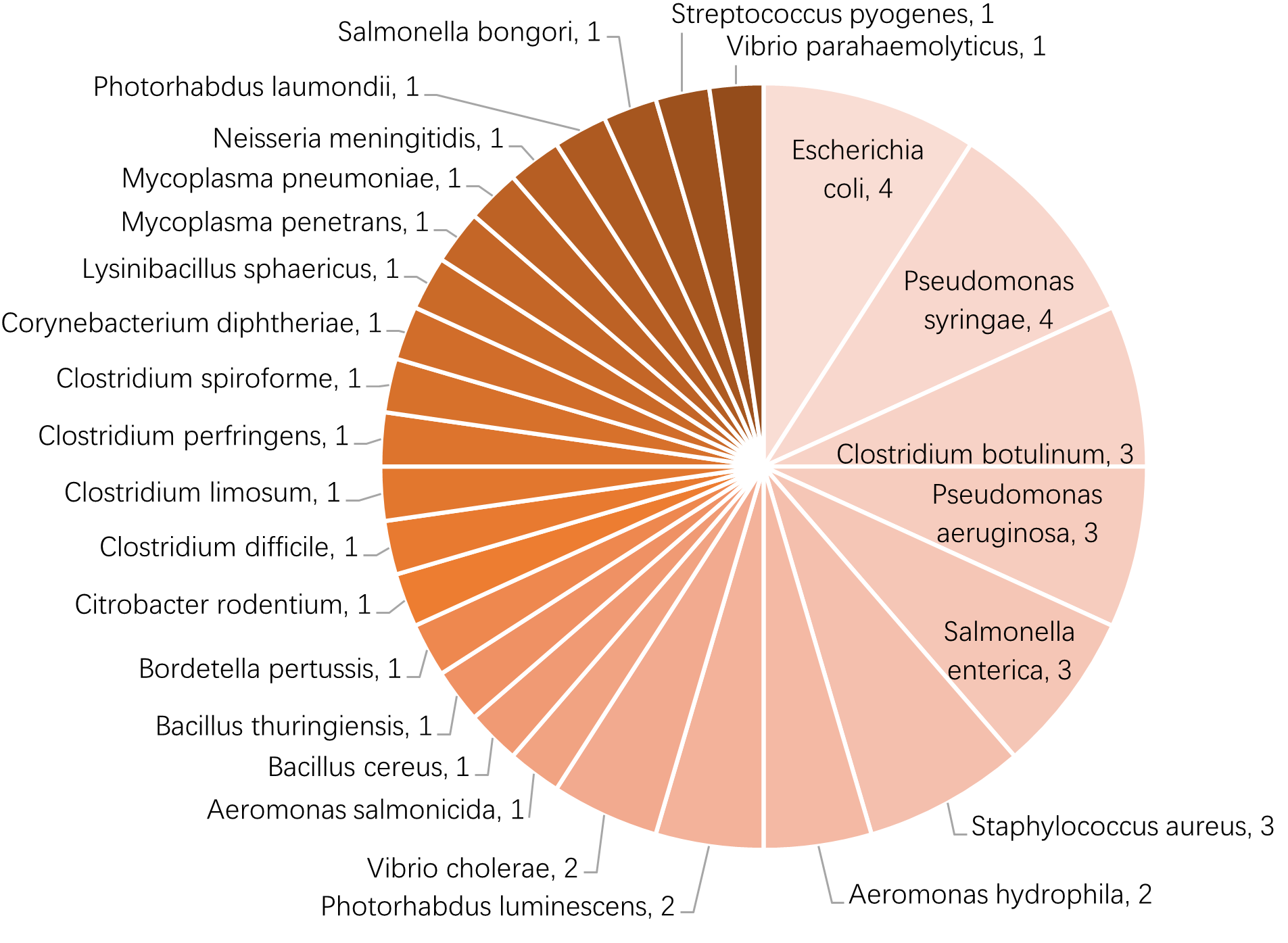

Supplement: btae378_Supplementary_Data [file btae378_supplementary_data.zip › Figure S2.tif]

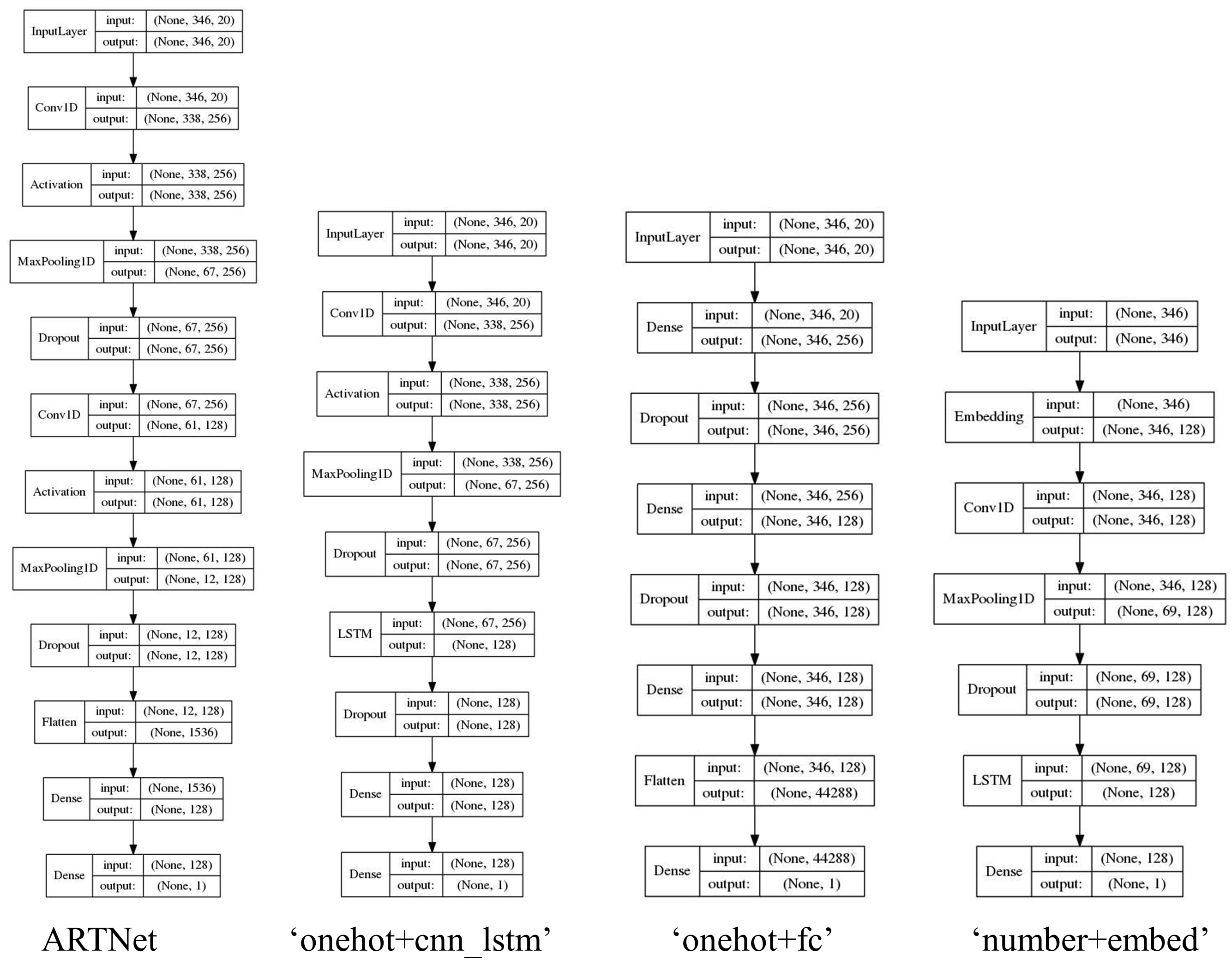

Supplement: btae378_Supplementary_Data [file btae378_supplementary_data.zip › Figure S5.tif]

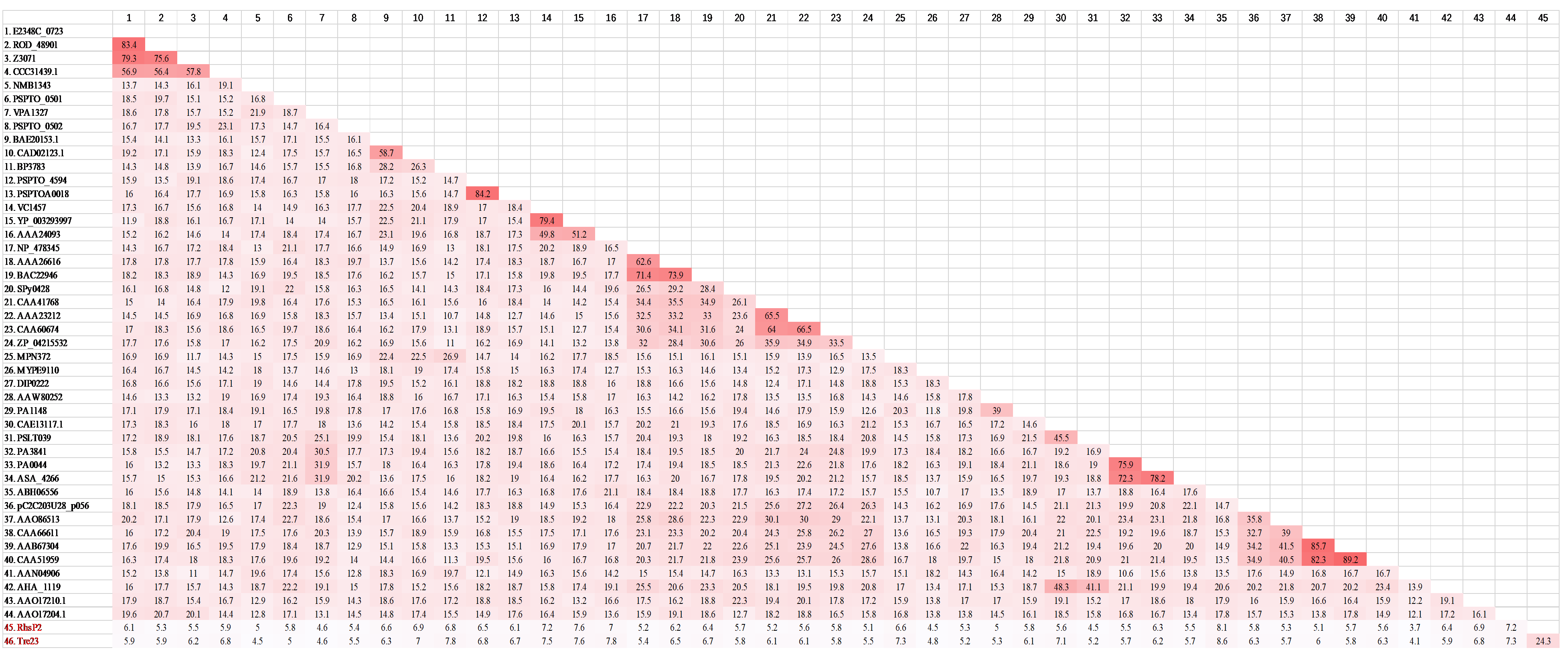

Supplement: btae378_Supplementary_Data [file btae378_supplementary_data.zip › Figure S1.tif]

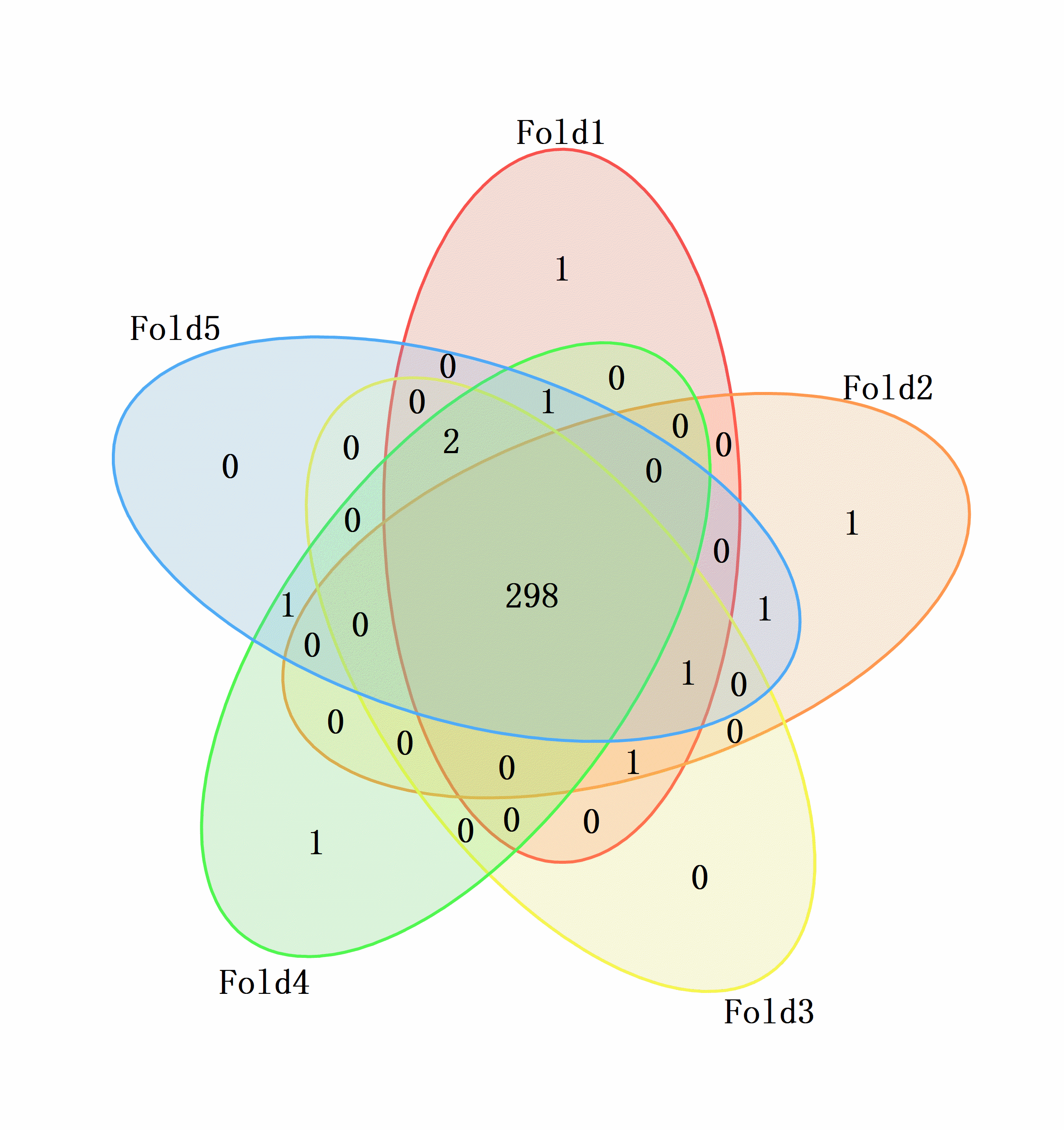

Supplement: btae378_Supplementary_Data [file btae378_supplementary_data.zip › Figure S7.tif]

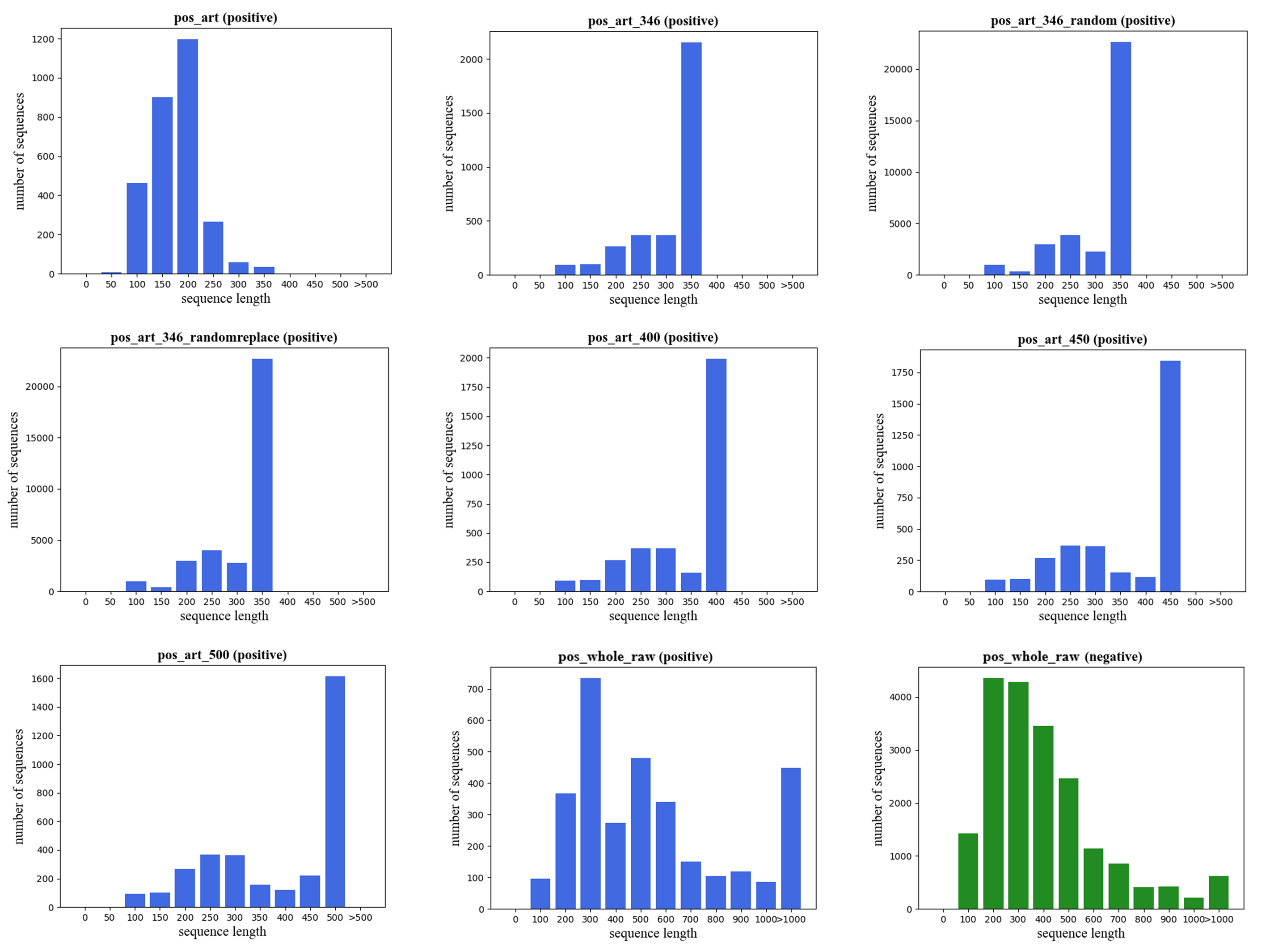

Supplement: btae378_Supplementary_Data [file btae378_supplementary_data.zip › Figure S4.tif]

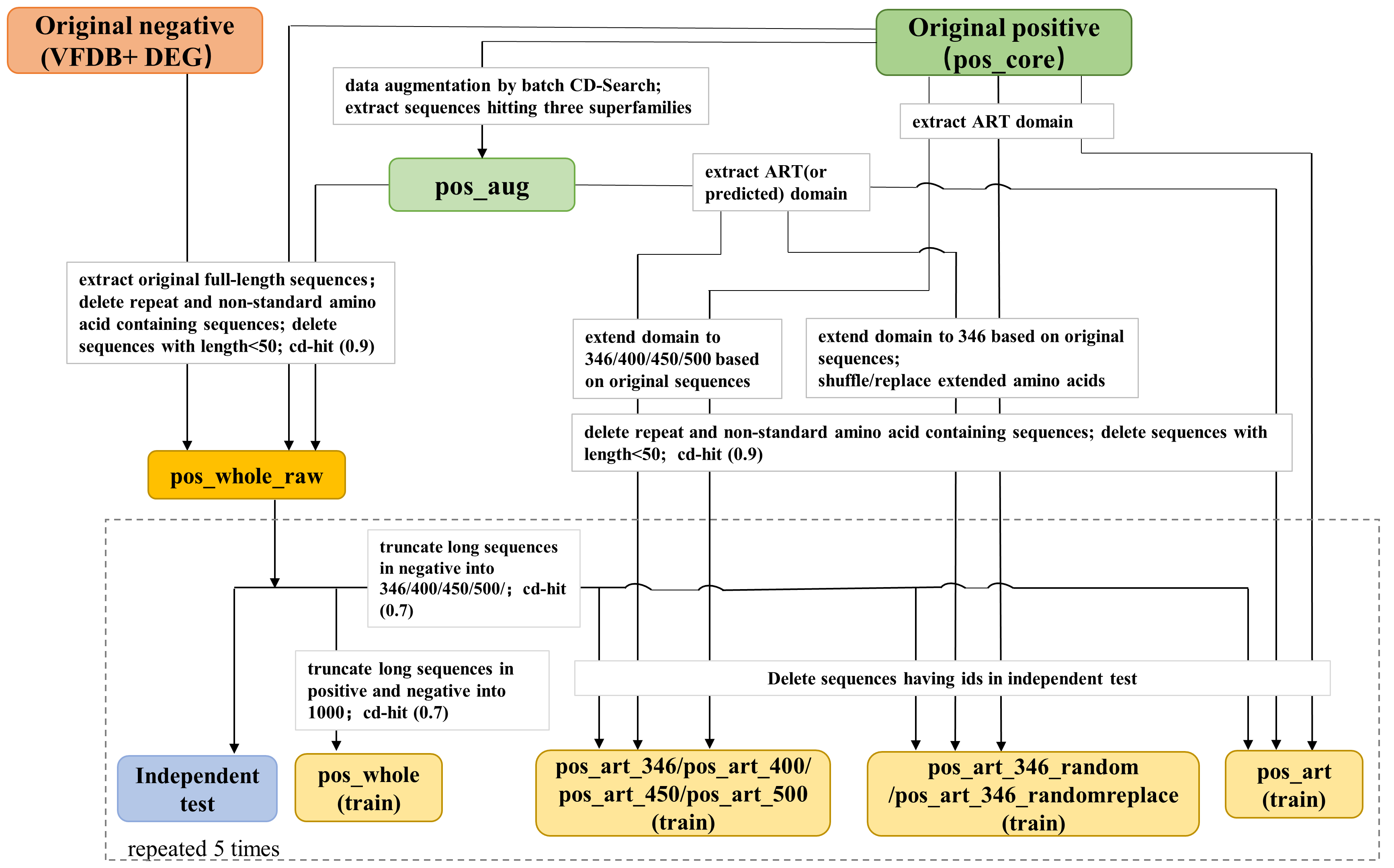

Supplement: btae378_Supplementary_Data [file btae378_supplementary_data.zip › Figure S3.tif]

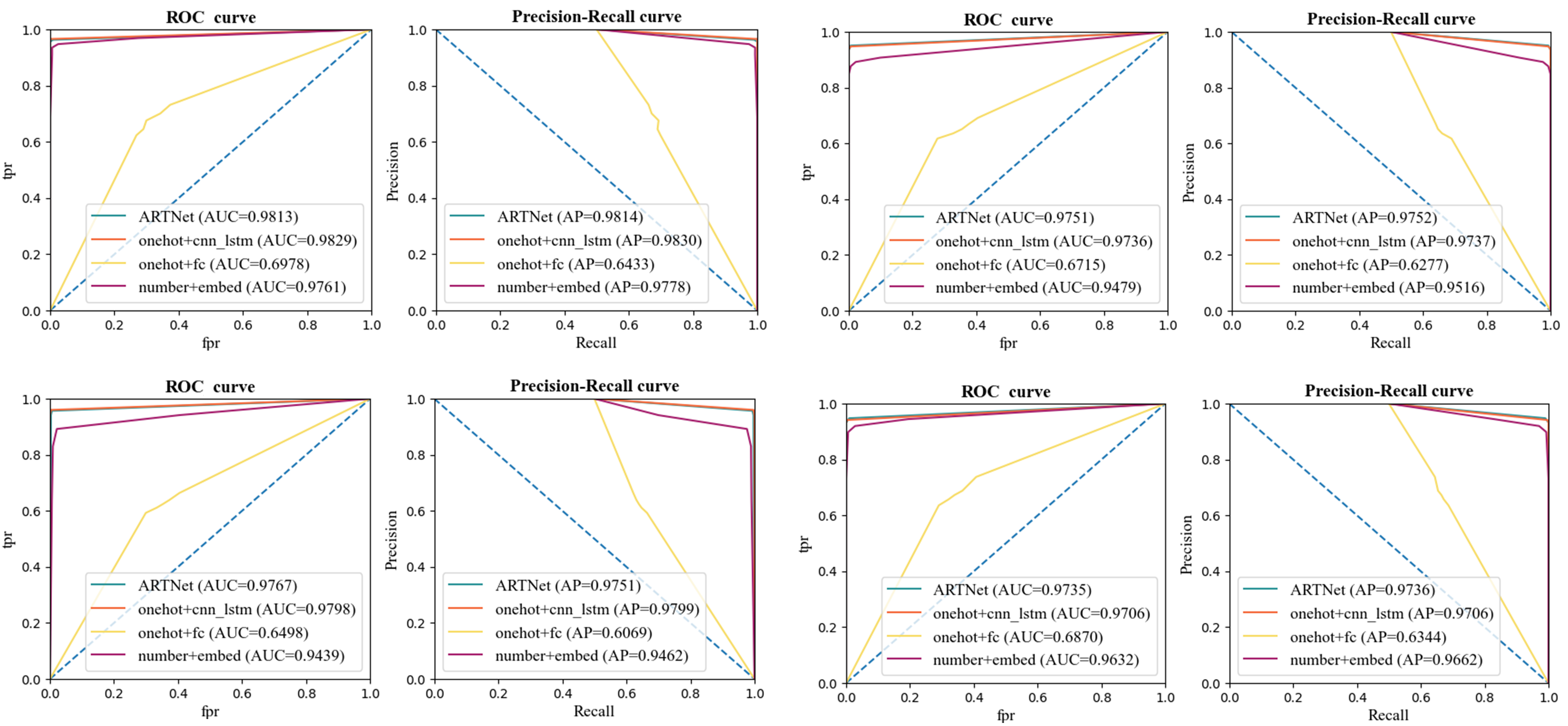

Supplement: btae378_Supplementary_Data [file btae378_supplementary_data.zip › Figure S8.tif]
